# An Interpretable Multimodal Model Integrating Multiparametric MRI Radiomics and Body Composition for Preoperative Risk Stratification of Malignant Transformation in Rectal Adenomas: A Two-Center Study

**DOI:** 10.3390/healthcare14213596

**Published:** 2026-10-23

**Authors:** Qiuhan Huang, Miaojia Yan, Yan Wu, Jieyi Cao, Yonggang Qiu, Peng Hu, Jihong Sun

**Affiliations:** 1 Department of Radiology, Sir Run Run Shaw Hospital, Zhejiang University School of Medicine, No. 3 East Qingchun Road, Shangcheng District, Hangzhou 310016, China; huangqiuhan@zju.edu.cn (Q.H.); 22518563@zju.edu.cn (M.Y.); jennifer_cao2023@163.com (J.C.); 2 Department of Radiology, The First Hospital of Jiaxing, No. 1882 Zhonghuan South Road, Nanhu District, Jiaxing 314001, China; 00227298@zjxu.edu.cn; 3 Department of Radiology, The First People’s Hospital of Xiaoshan District, 199 Shi Xin Nan Road, Xiaoshan District, Hangzhou 311200, China; bb46411545@sina.com

**Keywords:** rectal adenoma, malignant transformation, radiomics, body composition, machine learning

## Abstract

**Highlights:**

**What are the main findings?**

• The combined multimodal model, integrating high-resolution T2-weighted imaging (HRT2WI) and diffusion-weighted imaging (DWI) radiomics with clinical and body composition predictors, achieved AUCs of 0.956, 0.908, and 0.867 in the training, internal validation, and external validation cohorts, respectively, and outperformed the four component models and the junior radiologist in external validation.
• SHapley Additive exPlanations (SHAP) analysis identified the visceral-to-subcutaneous fat ratio, DWI radiomics model-predicted probability, and neutrophil-to-lymphocyte ratio as the leading contributors to the combined model, providing global and case-level interpretability.

**What are the implications of the main findings?**

• The interpretable model may support preoperative risk stratification of malignant transformation in rectal adenomas and help identify patients who warrant further diagnostic assessment or multidisciplinary discussion within established assessment pathways.
• Prospective multicenter validation, multi-reader assessment, and evaluation of clinical impact are required before the model can be considered for routine clinical use.

**Abstract:**

Background/Objectives: Preoperative identification of malignant transformation in rectal adenomas (RAs) is challenging because endoscopic biopsy may not sample focal malignant components. This study aimed to develop and externally validate an interpretable multimodal model for preoperative risk stratification of malignant transformation in RAs. Methods: In this retrospective two-center study, 359 surgically treated patients were allocated to training (*n* = 143), internal validation (*n* = 61), and external validation (*n* = 155) cohorts. Radiomic features derived from high-resolution T2-weighted imaging (HRT2WI) and diffusion-weighted imaging (DWI), clinical biomarkers, and CT-derived body composition metrics were evaluated. Five machine learning algorithms were compared for each radiomics signature. The final combined logistic regression model integrated the selected HRT2WI and DWI radiomics model-predicted probabilities with clinical and body composition predictors. Discrimination, calibration, and decision curve analyses were performed. Results: The malignant transformation was present in 65/143 patients (45.5%) in the training cohort and 91/155 (58.7%) in the external cohort, a difference of 13.3 percentage points. The combined model achieved area under the receiver operating characteristic curve (AUC) values of 0.956 (95% CI, 0.922–0.981), 0.908 (95% CI, 0.830–0.970), and 0.867 (95% CI, 0.803–0.919) in the training, internal validation, and external validation cohorts, respectively. In external validation, the combined model outperformed the four component models and the junior radiologist (all *p* < 0.05); its performance did not differ significantly from that of the senior radiologist (*p* = 0.093). Note that this reader comparison included only one junior and one senior radiologist and is exploratory and cannot support general conclusions regarding model performance versus radiologists in broader clinical practice. SHapley Additive exPlanations (SHAP) analysis identified the visceral-to-subcutaneous fat ratio (VSR), DWI radiomics model-predicted probability, and the neutrophil-to-lymphocyte ratio (NLR) as the leading contributors to the combined model. Conclusions: This interpretable two-center model showed promising discrimination for preoperative risk stratification of malignant transformation in surgically treated RAs. It may help identify patients who warrant further preoperative assessment or multidisciplinary discussion. Prospective multicenter evaluation is required before clinical implementation.

## 1. Introduction

Colorectal cancer (CRC) is the third most commonly diagnosed malignancy worldwide and remains a major cause of cancer mortality [1,2]. A substantial proportion of CRCs develop through the adenoma–carcinoma sequence, in which benign adenomatous epithelium accumulates molecular and histologic alterations before invasive cancer develops [3,4]. This prolonged pathway provides an opportunity for prevention; however, management is challenging when a malignant focus is small, heterogeneous, or located beneath an otherwise adenomatous surface. Colonoscopic biopsy may sample only superficial tissue and may therefore underestimate focal malignant transformation or depth of invasion [5,6]. A noninvasive method for preoperative risk stratification could help identify patients who may benefit from further diagnostic assessment or multidisciplinary discussion. Such a tool is intended to support established endoscopic, radiologic, and clinical assessment pathways rather than replace endoscopic assessment, pathologic diagnosis, or treatment decisions.

High-resolution T2-weighted imaging (HRT2WI) and diffusion-weighted imaging (DWI) provide complementary anatomic and diffusion information for evaluating malignant transformation in RAs [7,8,9]. However, visual assessment alone may not fully characterize intralesional heterogeneity. Radiomics extracts quantitative features from regions of interest and may capture imaging phenotypes that are not readily assessed visually [10,11,12]. Machine learning (ML) algorithms, including random forests and support vector machines, can model potentially complex associations among radiomic features [13,14]. The optimal algorithm for predicting malignant transformation in RAs remains uncertain, and previous studies have been limited by small samples and single-center development [8]. Imaging-only models may also omit systemic factors associated with malignant transformation.

In addition to lesion-associated characteristics, clinical inflammation and tumor marker profiles may provide complementary information. Serum carcinoembryonic antigen (CEA) has been associated with colorectal neoplasia, and the neutrophil-to-lymphocyte ratio (NLR) reflects systemic inflammation [15,16]. Quantitative CT studies have also linked visceral adipose tissue (VAT) to colorectal adenoma and advanced adenoma [17,18,19]. These observations support evaluating body composition alongside preoperative clinical and MRI information. However, the association between body composition and malignant transformation in RAs remains insufficiently characterized. Multimodal studies suggest that radiomics, body composition, and clinicopathologic variables may provide complementary predictive information [20,21,22].

Prior multimodal predictive studies for malignant transformation of RAs were mostly single center investigations without independent external validation; existing works also rarely incorporated CT derived body composition metrics alongside multiparametric MRI radiomics for risk stratification, and few provided model interpretability at both global and individual patient levels [8] (Table 1). Our primary testable hypothesis was that a multimodal model integrating HRT2WI- and DWI-derived radiomics-predicted probabilities, clinical inflammatory biomarkers, and CT-measured body composition indices would achieve superior discriminatory performance compared with single-modality models in an independent external validation cohort, while enabling interpretable risk output for preoperative clinical reference.

Therefore, this study aimed to develop and externally validate an interpretable multimodal model integrating HRT2WI and DWI radiomics signatures with clinical and body composition metrics for preoperative risk stratification of malignant transformation in RAs. The model was designed to provide a supportive risk stratification score that complements established assessment pathways and helps identify patients who may warrant further diagnostic assessment or multidisciplinary discussion; it was not designed as a standalone diagnostic or treatment decision tool.

## 2. Materials and Methods

### 2.1. Patients

**Figure 1:**
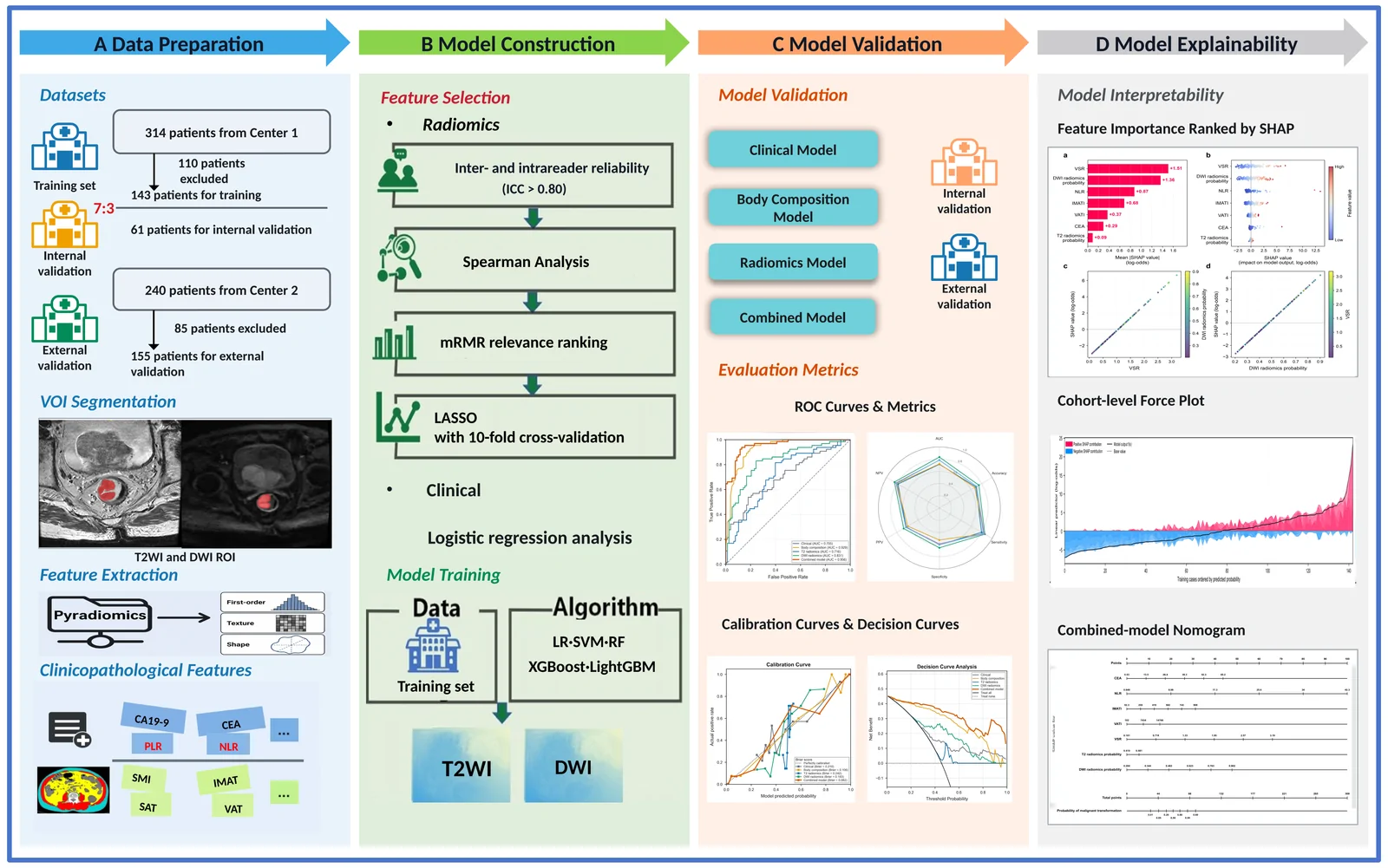
Study workflow. (**A**) Data preparation: two cohorts were assembled; high-resolution T2-weighted imaging (HRT2WI) and diffusion-weighted imaging (DWI) regions of interest (ROIs) were delineated; HRT2WI and DWI radiomic features were extracted; and clinical factors and body composition metrics were collected. (**B**) Model construction: radiomic features were selected separately for the HRT2WI and DWI radiomics models, and clinical factors and body composition predictors were integrated into predictive models using five machine learning algorithms. (**C**) Model validation: clinical, body composition, radiomics, and combined models were evaluated in internal and external validation cohorts using area under the receiver operating characteristic curve (AUC), calibration curves, and decision curve analysis. (**D**) Model interpretability: SHAP analysis and a nomogram were used to evaluate feature importance and contributions to the model output.

This retrospective two-center study included patients who underwent rectal surgery and had pathologically confirmed RA or malignant transformation of RA between June 2016 and June 2024 at two independent hospitals (Center 1: Sir Run Run Shaw Hospital, Zhejiang University School of Medicine, China; Center 2: The Second Affiliated Hospital Zhejiang University School of Medicine, China). The overall study workflow is shown in Figure 1. The inclusion criteria were as follows: (1) postoperative pathologic confirmation of RA or malignant transformation of RA; (2) pelvic MRI, including HRT2WI and DWI, and abdominal CT performed within 2 weeks before surgery; and (3) complete clinicopathologic data. The exclusion criteria were as follows: (1) preoperative neoadjuvant therapy; (2) a history of other malignant tumors; (3) a tumor with a maximum diameter of less than 10 mm that could not be accurately delineated; and (4) poor image quality or substantial artifacts. The numbers of included and excluded patients are provided in Supplementary Figure S1. Patients from Center 1 (*n* = 204) were randomly divided into training (*n* = 143) and internal validation (*n* = 61) cohorts at a 7:3 ratio. Patients from Center 2 (*n* = 155) served as the independent external validation cohort. The institutional review boards of the participating centers approved this retrospective multicenter study and waived the requirement for written informed consent.

### 2.2. Clinicopathological Data

Clinical data were collected from electronic medical records. Candidate variables were limited to preoperative factors available at both centers: sex, age, hypertension, diabetes, smoking, alcohol use, family history, body mass index (BMI), carcinoembryonic antigen (CEA), carbohydrate antigen 19-9 (CA19-9), carbohydrate antigen 125 (CA125), white blood cell count (WBC), red blood cell count (RBC), hemoglobin, platelet count, neutrophil count, lymphocyte count, monocyte count, neutrophil-to-lymphocyte ratio (NLR), platelet-to-lymphocyte ratio (PLR), lymphocyte-to-monocyte ratio (LMR), and hemoglobin-to-platelet ratio (HPR). NLR, PLR, LMR, and HPR were recalculated from the source blood counts. Histopathologic results for surgically resected specimens were extracted from electronic medical records. Patients were categorized as having RA or malignant transformation of RA according to postoperative pathologic findings. RA was defined as mucosal epithelial dysplasia and was categorized as low-grade intraepithelial neoplasia (LGIN) or high-grade intraepithelial neoplasia (HGIN) according to epithelial architectural and cytologic atypia. Malignant transformation of RA was defined as focal malignant morphologic transformation arising within dysplastic adenomatous tissue, characterized by enlarged round-to-oval nuclei, coarse chromatin, prominent nucleoli, and pathologic mitotic figures.

### 2.3. CT Body Composition Measurements

Body composition parameters were measured on transverse noncontrast CT images at the third lumbar vertebral (L3) level using SliceOMatic V5.0 (TomoVision, Magog, QC, Canada) and tissue-specific Hounsfield unit (HU) thresholds [24]: −29 to +150 HU for skeletal muscle, −150 to −50 HU for visceral adipose tissue, and −190 to −30 HU for subcutaneous and intermuscular adipose tissue (Supplementary Figure S2). Skeletal muscle area (SMA, cm^2^), visceral adipose tissue area (VAT, cm^2^), subcutaneous adipose tissue area (SAT, cm^2^), and intermuscular adipose tissue area (IMAT, cm^2^) were normalized to squared lumbar height (Hlumbar^2^) to derive the skeletal muscle index (SMI, cm^2^/m^2^), visceral adipose tissue index (VATI, cm^2^/m^2^), subcutaneous adipose tissue index (SATI, cm^2^/m^2^), and intermuscular adipose tissue index (IMATI, cm^2^/m^2^). Hlumbar was used for normalization because it correlated with stature and was available from abdominal images when anthropometric height data were unavailable [25,26]. The visceral-to-subcutaneous fat ratio (VSR) was calculated by dividing VATI by SATI. Skeletal muscle density (SMD), a proxy for muscle quality, was also measured. Sex-specific cutoffs were derived only in the training cohort by receiver operating characteristic (ROC) analysis using the maximum Youden index (Supplementary Figures S3 and S4) and were then applied unchanged to the internal and external validation cohorts.

### 2.4. Region of Interest Segmentation and Feature Extraction

All patients underwent preoperative 3.0-T MRI with an eight-channel phased-array body coil in the supine position within 2 weeks before surgery. HRT2WI and DWI were selected for lesion segmentation. Detailed scanner information and imaging parameters are provided in Supplementary Table S1. Tumor regions of interest (ROIs) were manually delineated slice by slice along the tumor margins on HRT2WI and DWI using ITK-SNAP (version 4.2.0; University of Pennsylvania, Philadelphia, PA, USA, www.itksnap.org) by a radiologist with 5 years of abdominal imaging experience. Two senior radiologists with more than 10 years of experience reviewed the delineations, and final contours were established by consensus. Both sequences underwent the sequence-specific preprocessing pipelines described in the Supplementary Materials before feature extraction. PyRadiomics (version 3.0.1) was used to extract original-image features only. No additional resampling or intensity normalization was performed during feature extraction, and the bin width was 0.25. A total of 107 radiomic features were derived from each HRT2WI and DWI ROI: 14 shape-based features, 18 first-order features, 24 gray-level co-occurrence matrix features, 16 gray-level run-length matrix features, 16 gray-level size zone matrix features, 14 gray-level dependence matrix features, and 5 neighboring gray-tone difference matrix features.

### 2.5. Feature Selection and Radiomics Model Construction

To assess radiomic feature reproducibility, one radiologist repeated segmentation in 30 randomly selected cases sampled exclusively from the training cohort after 1 month. Intraobserver reproducibility was assessed using intraclass correlation coefficients (ICCs). Features with an ICC of at least 0.80 were retained for subsequent analysis. Spearman correlation analysis was then used to remove highly redundant features; variables with a correlation coefficient greater than 0.90 were eliminated. The maximum relevance minimum redundancy (mRMR) algorithm was applied, and the top-ranked 20 features according to standard mRMR objective score were retained for downstream LASSO regression. The mRMR ranks features by optimizing mutual information-based relevance to endpoint and minimal redundancy among candidate features. Least absolute shrinkage and selection operator (LASSO) regression was then applied for further dimensionality reduction, with 10-fold cross-validation used to identify the optimal regularization parameter, λ. All feature reproducibility filtering (ICC), spearman correlation pruning, mRMR ranking and LASSO dimensionality reduction steps were strictly confined exclusively to the training cohort. No data from internal or external validation cohorts were used at any feature selection stage to avoid data leakage; validation cohorts were only used for final locked model performance assessment. After the final feature sets were determined, 1000 bootstrap resamples were used to record the frequency with which each final feature received a nonzero LASSO coefficient. Bootstrap selection frequencies were used descriptively to characterize the sensitivity of feature selection to perturbations of the training sample. Given only 65 malignant transformation events in the training cohort, bootstrap resampling also served to appraise feature selection stability; wide variation in bootstrap selection frequency would indicate unstable feature choice. No minimum frequency threshold was prespecified, and bootstrap results were not used to remove or replace the locked final features. The results of 1000 bootstrap resamples are presented in the Supplementary Figure S5. Separate HRT2WI and DWI radiomics models were constructed using the selected radiomic features. Five ML algorithms—logistic regression (LR), support vector machine (SVM), random forest (RF), Extreme Gradient Boosting (XGBoost), and Light Gradient Boosting Machine (LightGBM)—were tuned in the training cohort using stratified five-fold cross-validation to identify the best-performing classifier.

To assess selection optimism induced by sequential rounds of feature screening, hyperparameter tuning, and comparison among five algorithms, supplementary five-fold outer/five-fold inner nested cross-validation was performed separately for HRT2WI and DWI. Within each outer training subset, outcome dependent correlation pruning, candidate feature selection, LASSO fitting, algorithm tuning, and algorithm selection were fully replicated using training subset only data. Outer held out observations were used exclusively for model performance evaluation. Patient level outer fold predictions were pooled to obtain discrimination performance that accounts for the entire feature selection workflow. This supplementary analysis was implemented solely to evaluate the complete modeling pipeline and was not used to retroactively replace the original primary analysis models.

### 2.6. Construction of the Clinical, Body Composition, and Combined Models

In addition to the radiomics models, clinical, body composition, and combined models were developed. Clinical variables associated with malignant transformation in univariable logistic regression (*p* < 0.05) were entered into an initial multivariable logistic regression model. Backward elimination sequentially removed the variable with the largest Wald *p* value until all retained variables met the *p* < 0.05 criterion, yielding a parsimonious clinical model. For body composition analysis, risk-oriented strata associated with malignant transformation in univariable analysis were screened for redundancy. When the absolute Spearman correlation coefficient was at least 0.80, the variable with the smaller univariable *p* value was retained. The final variables after redundancy reduction were entered simultaneously into the locked multivariable body composition model. Because univariable screening and backward elimination may introduce selection instability and optimism, the original clinical and body composition models were retained as the primary analyses, and alternative Elastic Net–penalized models were evaluated as sensitivity analyses. Before fitting the revised models, five clinical candidate variables (age, sex, CEA, CA19-9, and NLR) and five body composition candidate variables (SMI, SMD, IMATI, VATI, and SATI) were predefined based on the previous literature [15,16,19,22] together with clinical and measurement considerations. Separate Elastic Net–penalized logistic regression models were built without univariable *p*-value screening or post-selection unpenalized refitting. Variable standardization and hyperparameter tuning were performed only within training folds in the 5 × 5 nested cross-validation. The final revised models were fitted on the training cohort and applied identically to the internal and external validation cohorts. The final combined model integrated these independent predictors with the HRT2WI and DWI radiomics model-predicted probabilities using logistic regression. Model development and tuning were conducted in the training cohort; the resulting model specifications and operating thresholds were fixed before evaluation in the internal and external validation cohorts. The model generated a continuous preoperative risk-stratification score. Higher scores were intended to identify patients who may warrant further diagnostic assessment or multidisciplinary discussion in conjunction with routine endoscopic, radiologic, and clinical findings. Discrimination, calibration, and potential clinical utility were assessed in all cohorts using ROC AUCs, calibration curves, and decision curve analysis (DCA), respectively. SHapley Additive exPlanations (SHAP) quantified global and individual feature contributions on the log-odds scale. A nomogram was derived from the final logistic regression score to provide a graphical representation for preoperative risk stratification.

### 2.7. Reader Study

A reader study compared the discrimination of the combined model with that of radiologists for classifying RA and malignant transformation of RA. One junior radiologist with 3 years of experience and one senior radiologist with 10 years of experience independently interpreted MRI and contrast-enhanced CT examinations while blinded to pathologic diagnoses. Prespecified assessment domains included tumor size, morphology, signal and enhancement characteristics, adjacent rectal wall changes, relationships with surrounding structures, regional lymph node features, stenosis, and distant metastatic signs. The readers’ diagnostic assessments were recorded separately, and their ROC curves were compared with that of the combined model.

### 2.8. Statistical Analysis

Descriptive statistical analyses were performed using R (version 4.3.3, R Core Team, Vienna, Austria) and Python (version 3.10, Python Software Foundation, Wilmington, DE, USA). The distribution of continuous variables was assessed using Shapiro–Wilk tests, histograms, and Q–Q plots. Normally distributed variables were presented as mean ± standard deviation, and non-normally distributed variables were presented as median with interquartile range. Within each cohort, continuous variables were compared using Welch’s *t* tests or Mann–Whitney U tests, as appropriate. Cross-cohort continuous comparisons were performed using Welch’s ANOVA or Kruskal–Wallis tests. Categorical variables were summarized as frequencies and percentages and compared using Pearson’s chi-square test or Fisher–Freeman–Halton exact test, as appropriate. The Benjamini–Hochberg false discovery rate procedure was applied for multiple comparison correction, and both unadjusted *p* values and adjusted *q* values were reported. Model performance was evaluated in terms of discrimination, calibration, and clinical utility. Discrimination was quantified by ROC AUCs with 95% confidence intervals, accuracy, sensitivity, specificity, positive predictive value, and negative predictive value. For each model, the classification threshold was selected by maximizing the Youden index in the training cohort and was applied unchanged to the internal and external validation cohorts when calculating accuracy, sensitivity, specificity, PPV, and NPV. Calibration was assessed using calibration curves. Decision curve analysis was used to evaluate clinical net benefit. The DeLong test was performed for pairwise comparison of AUCs. All statistical tests were two-sided, with *p* < 0.05 indicating nominal statistical significance and *q* < 0.05 indicating statistical significance after multiplicity adjustment.

## 3. Results

### 3.1. Patient Characteristics

A total of 359 patients met the inclusion criteria. The training, internal validation, and external validation cohorts comprised 143 patients (mean age, 66 ± 12 years; 77 men), 61 patients (mean age, 65 ± 11 years; 34 men), and 155 patients (mean age, 63 ± 11 years; 96 men), respectively. Malignant transformation occurred in 45.5% (65 of 143), 45.9% (28 of 61), and 58.7% (91 of 155) of patients in the training, internal validation, and external validation cohorts, respectively. The malignant transformation rate differed significantly across the three cohorts (overall *p* = 0.048; Supplementary Table S2), with a higher proportion of malignant transformation in the external validation cohort than in the training and internal validation cohorts. Across-cohort comparisons showed nominal differences in diabetes, smoking history, alcohol use, CEA, CA19-9, neutrophil count, and NLR (all unadjusted *p* < 0.05; Table 2); only CEA and CA19-9 remained significant after Benjamini–Hochberg false discovery rate adjustment (both *q* < 0.001; Table 2). In the training cohort, the malignant transformation group had higher CEA, CA19-9, and NLR and lower lymphocyte counts and LMR than the RA group; these differences remained significant after adjustment (*q* = 0.028, 0.006, 0.028, 0.037, and 0.007, respectively; Table 2). Hemoglobin and PLR showed nominal between-group differences but did not remain significant after adjustment (*q* = 0.150 and 0.091, respectively; Table 2).

### 3.2. Construction of the Clinical and Body Composition Models

In the training cohort, univariable logistic regression identified CEA, CA19-9, hemoglobin, lymphocyte count, monocyte count, NLR, PLR, and LMR as candidate predictors of malignant transformation (all *p* < 0.05; Table 3). Backward multivariable logistic regression retained CEA (odds ratio [OR], 1.228; 95% CI, 1.055–1.429; *p* = 0.008) and NLR (OR, 1.244; 95% CI, 1.059–1.463; *p* =0.008). The clinical model achieved AUCs of 0.705 (95% CI, 0.623–0.788), 0.669 (95% CI, 0.518–0.804), and 0.668 (95% CI, 0.574–0.758) in the training, internal validation, and external validation cohorts, respectively. All 10 sex-specific body composition strata were associated with malignant transformation in univariable analysis (all *p* < 0.05; Table 4). After redundancy reduction, eight variables entered the multivariable model. IMATI (OR, 6.79; 95% CI, 1.50–30.73; *p* = 0.013), VATI (OR, 10.70; 95% CI, 1.27–89.91; *p* = 0.029), and VSR (OR, 4.91; 95% CI, 1.04–23.09; *p* = 0.044) were independently associated with malignant transformation. The body composition model achieved AUCs of 0.929 (95% CI, 0.886–0.965), 0.804 (95% CI, 0.674–0.910), and 0.809 (95% CI, 0.740–0.875) in the training, internal validation, and external validation cohorts, respectively (Table 5).

In supplementary sensitivity analyses using Elastic Net regression, all five clinical candidates and four of the five body composition candidates were retained. The sensitivity analyses clinical and body composition models achieved external validation AUCs of 0.691 (95% CI, 0.593–0.770) and 0.870 (95% CI, 0.814–0.923), respectively. Full performance results, calibration plots, and decision curve analyses are presented in the Supplementary Figure S6.

### 3.3. Feature Selection and Radiomics Signature Building

**Figure 2:**
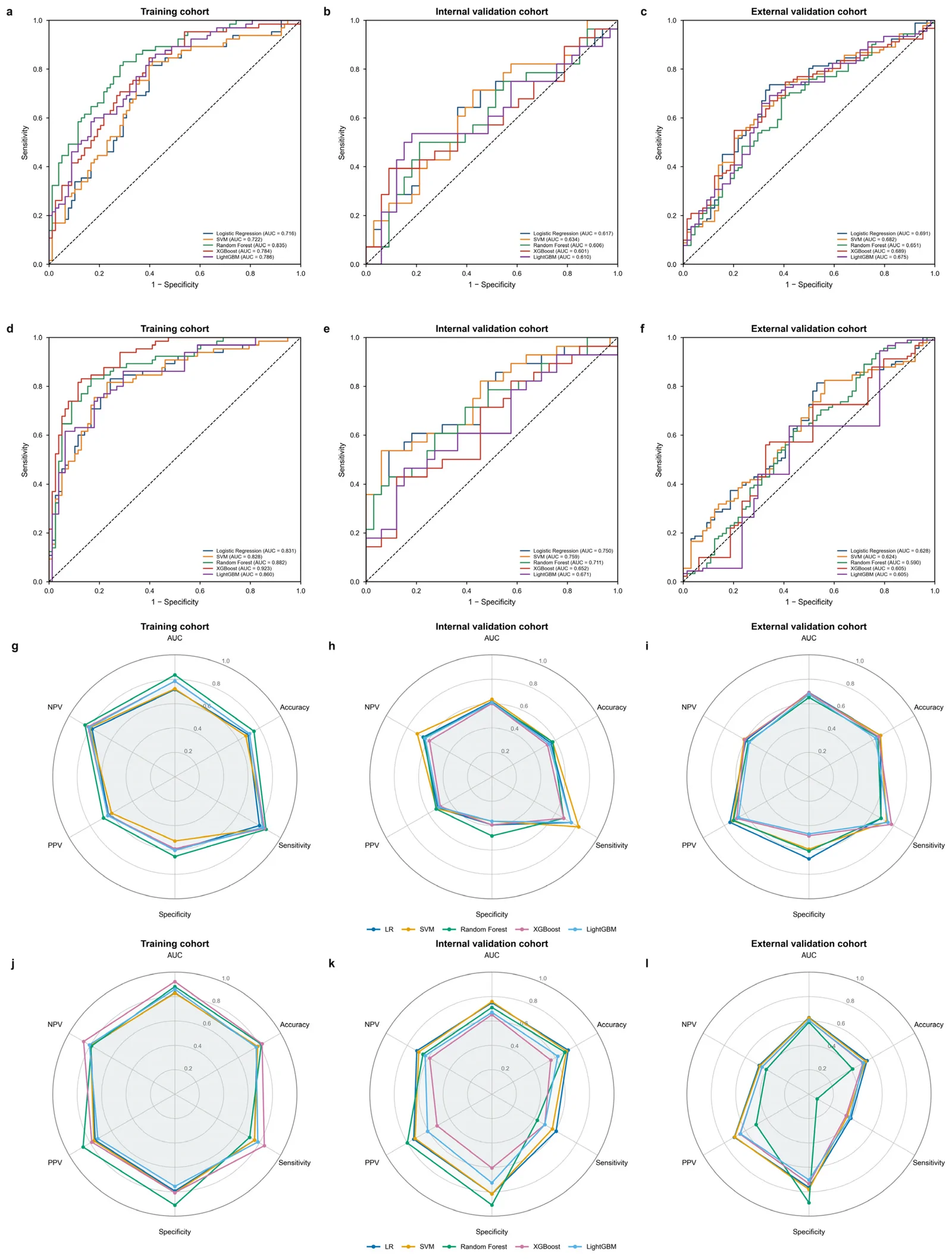
Receiver operating characteristic (ROC) curves and radar charts comparing the performance of five machine learning algorithms for high-resolution T2-weighted imaging (HRT2WI) and diffusion-weighted imaging (DWI) radiomics models in the training, internal validation, and external validation cohorts. (**a**–**c**) ROC curves for LR, SVM, RF, XGBoost, and LightGBM HRT2WI radiomics models. (**d**–**f**) ROC curves for LR, SVM, RF, XGBoost, and LightGBM DWI radiomics models. (**g**–**i**) Radar charts for HRT2WI radiomics models. (**j**–**l**) Radar charts for DWI radiomics models. AUC = area under the curve; PPV = positive predictive value; NPV = negative predictive value; LR = logistic regression; SVM = support vector machine; RF = random forest; XGBoost = Extreme Gradient Boosting; LightGBM = Light Gradient Boosting Machine.

Of the 107 extracted features, 97 HRT2WI and 84 DWI radiomic features met the ICC criterion (ICCs > 0.80). After Spearman correlation pruning, 44 HRT2WI and 33 DWI candidate features remained. mRMR ranking followed by LASSO logistic regression reduced each sequence to seven features for radiomics model construction. The LASSO selection paths and feature coefficients are shown in Supplementary Figure S7 and S8. Five machine learning algorithms (LR, SVM, RF, XGBoost, and LightGBM) were then compared using the selected HRT2WI and DWI feature sets. For HRT2WI, LR showed the most consistent discrimination across cohorts, with AUCs of 0.716 (95% CI, 0.629–0.800), 0.617 (95% CI, 0.477–0.763), and 0.691 (95% CI, 0.606–0.776) in the training, internal validation, and external validation cohorts, respectively (Figure 2; Supplementary Table S3). The mean five-fold cross-validation AUC was 0.713 ± 0.079, and the training cohort threshold was 0.493. For DWI, LR was likewise selected as the base classifier, achieving AUCs of 0.831 (95% CI, 0.760–0.895), 0.750 (95% CI, 0.617–0.869), and 0.628 (95% CI, 0.534–0.717) across the three cohorts, respectively (Figure 2; Supplementary Table S4). The mean five-fold cross-validation AUC was 0.817 ± 0.047, and the training cohort threshold was 0.455. LR was selected based on its discrimination in five-fold cross-validation within the training cohort, together with its parsimonious model structure and interpretability. Given the limited training sample size, LR provided a relatively simple approach to estimating probabilities for subsequent multimodal integration. Neither the internal nor the external validation cohort was used for algorithm selection. The predicted probabilities from the LR-based HRT2WI and DWI models were subsequently incorporated into the combined model. As a supplementary sensitivity analysis, the radiomics model development strategy was evaluated using selection-aware nested cross-validation. The pooled outer-fold AUCs were 0.518 for HRT2WI and 0.783 for DWI (Supplementary Table S5).

### 3.4. Construction and Comparison of the Combined Model

**Figure 3:**
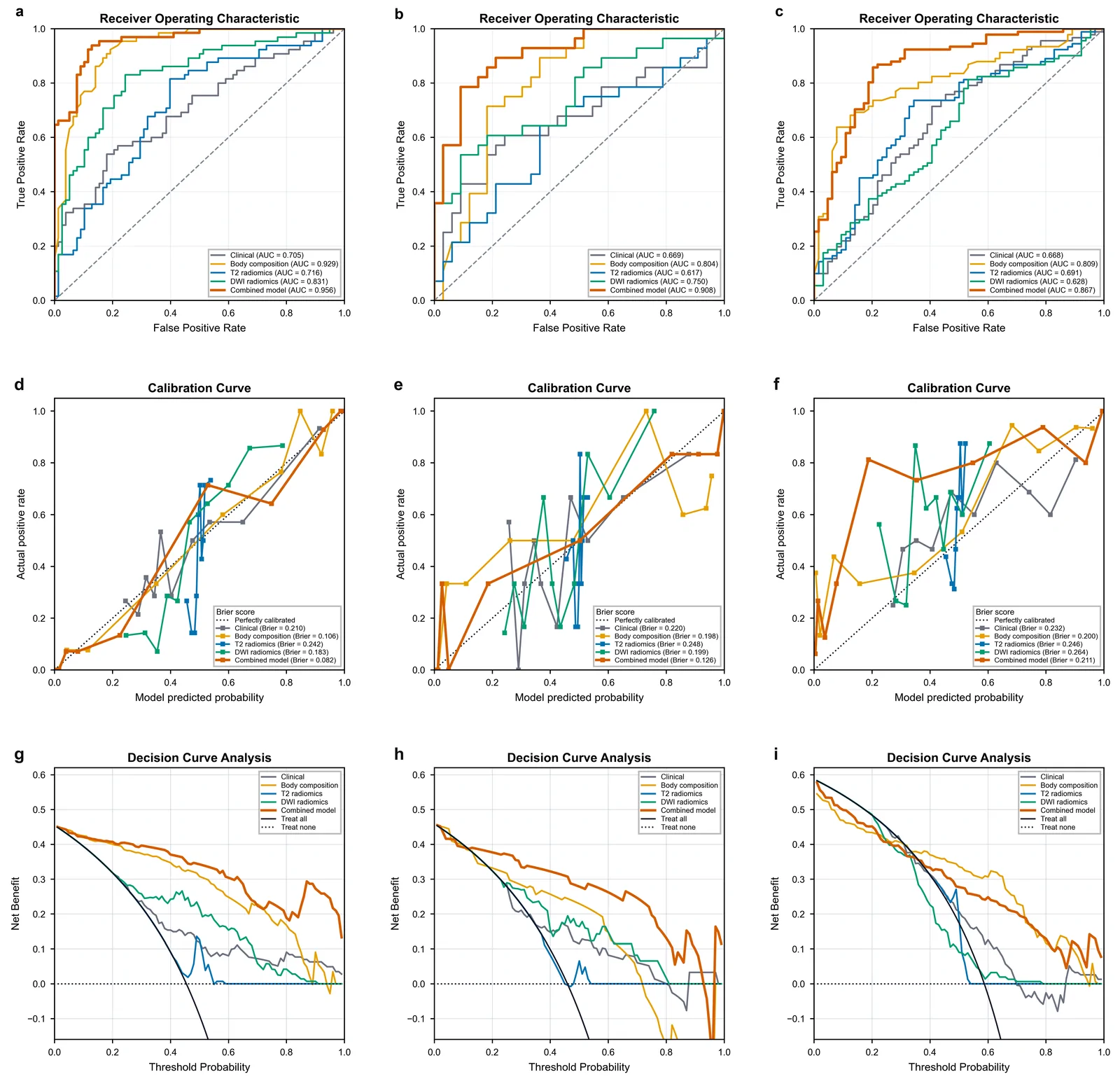
Performance of clinical, body composition, HRT2WI radiomics, DWI radiomics, and combined models for predicting malignant transformation of rectal adenomas. (**a**–**c**) Receiver operating characteristic (ROC) curves for the training, internal validation, and external validation cohorts, respectively. (**d**–**f**) Calibration curves for the training, internal validation, and external validation cohorts, respectively. (**g**–**i**) Decision curves for the training, internal validation, and external validation cohorts, respectively.

**Figure 4:**
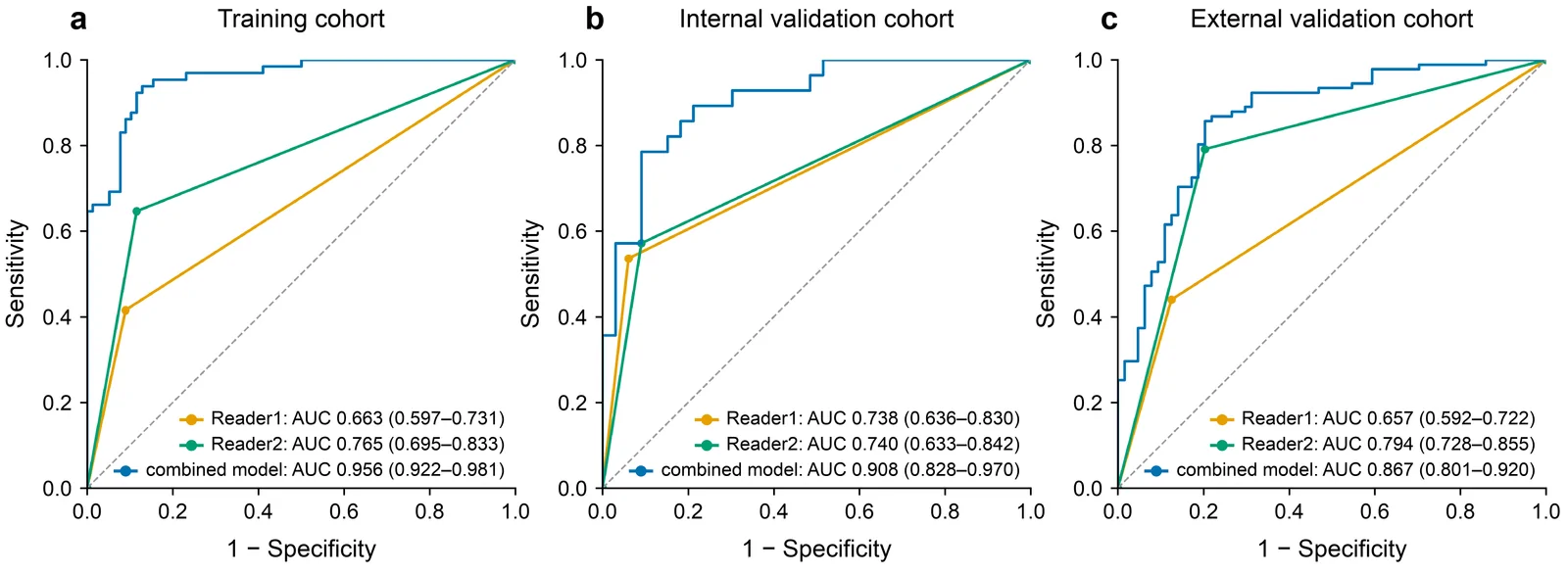
Receiver operating characteristic (ROC) curves comparing the combined model with two radiologists for predicting malignant transformation of rectal adenomas. ROC analyses were performed in the training (**a**), internal validation (**b**), and external validation (**c**) cohorts. Curves are shown for the combined model (blue), Reader 1 (orange), and Reader 2 (green). Corresponding AUCs and 95% confidence intervals are labeled. The gray dashed line indicates the non-discrimination reference line.

The combined model integrated the HRT2WI and DWI radiomics model-predicted probabilities with CEA, NLR, IMATI, VATI, and VSR. It yielded AUCs of 0.956 (95% CI, 0.922–0.981), 0.908 (95% CI, 0.830–0.970), and 0.867 (95% CI, 0.803–0.919) in the training, internal validation, and external validation cohorts, respectively (Table 5; Figure 3a–c). Accuracy, sensitivity, specificity, positive predictive value, and negative predictive value for all five models are summarized in Table 5. In the validation cohorts, the combined model had higher AUCs than each component model (paired DeLong tests, all *p* < 0.05; Table 5). Calibration curves and decision curves for all models are shown in Figure 3d–i. In external validation, the combined model had a calibration intercept of 1.207 (95% CI, 0.728–1.967), a slope of 0.619 (95% CI, 0.445–0.900), and a Brier score of 0.211 (95% CI, 0.168–0.262). The mean predicted risk was lower than the observed event proportion (0.394 vs. 0.587), indicating that absolute risk estimates require cautious interpretation and further recalibration before clinical use. Decision curve analysis showed that the combined model’s net benefit varied across cohorts and threshold probabilities; paired net benefit differences with 95% CIs are reported in Supplementary Table S6. The junior radiologist’s AUCs were 0.663 (95% CI, 0.597–0.731), 0.738 (95% CI, 0.636–0.830), and 0.657 (95% CI, 0.592–0.722) in the training, internal validation, and external validation cohorts, respectively. The senior radiologist’s AUCs were 0.765 (95% CI, 0.695–0.833), 0.740 (95% CI, 0.633–0.842), and 0.794 (95% CI, 0.728–0.855), respectively (Figure 4). In the external validation cohort, the combined model outperformed the junior radiologist (0.867 vs. 0.657; Holm-adjusted *p* < 0.001), whereas the difference from the senior radiologist was not statistically significant (0.867 vs. 0.794; Holm-adjusted *p* = 0.093). This exploratory comparison involved merely one junior and one senior radiologist; it does not constitute evidence that the model generally outperforms radiologists, and multi-reader studies are required for further assessment.

### 3.5. Model Interpretability

**Figure 5:**
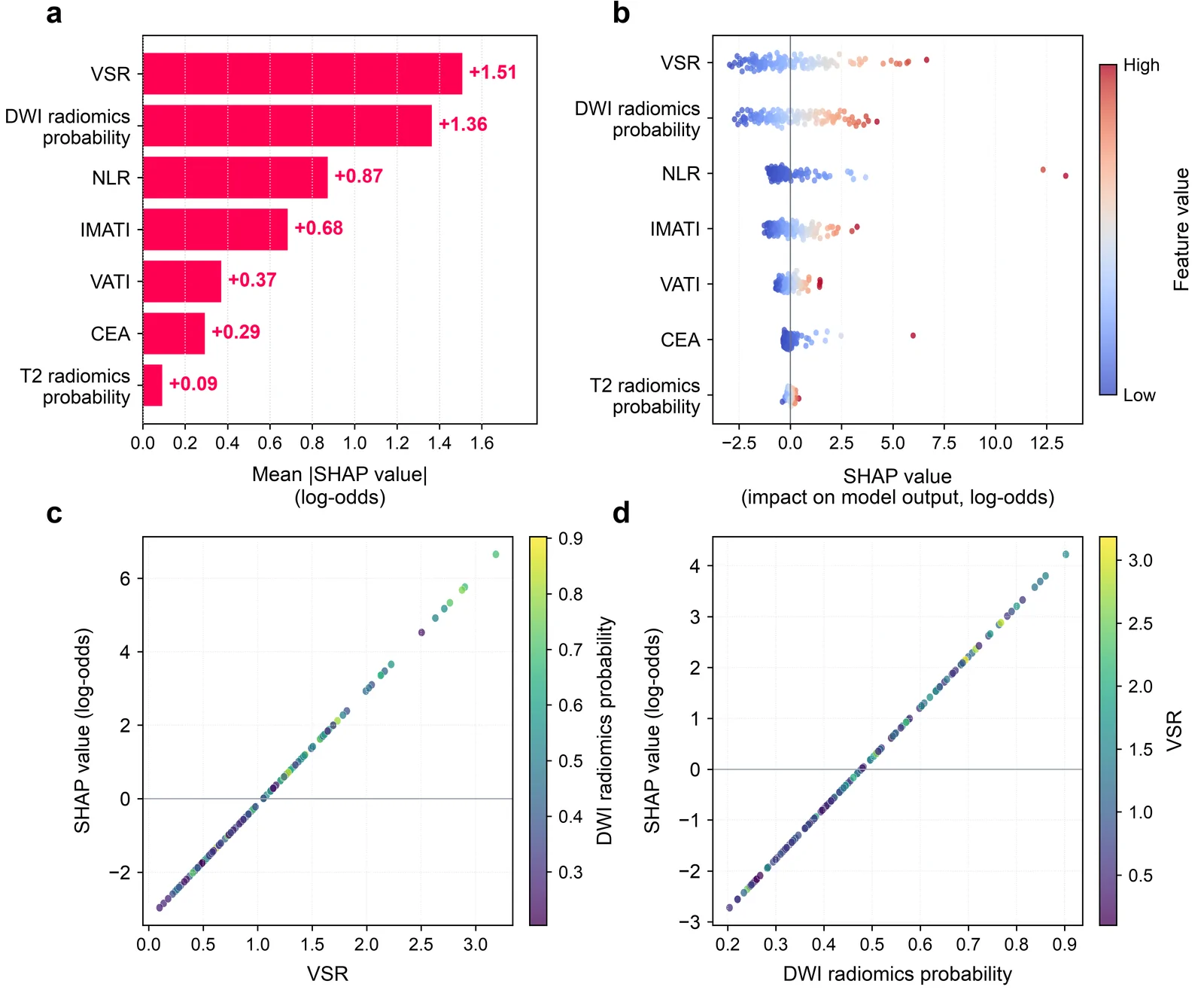
Global SHAP-derived interpretability of the multimodal prediction model. (**a**) Bar plot of mean absolute SHAP values on the log-odds scale, ranking the overall predictive importance of each feature. VSR was the most influential predictor. (**b**) Beeswarm summary plot of SHAP values for individual patients. Each dot represents one sample; dot color corresponds to the original feature value (blue = low, red = high). Positive SHAP values increase the predicted risk of malignant transformation. (**c**,**d**) SHAP dependence plots showing the marginal associations of VSR (**c**) and DWI radiomics model-predicted probability (**d**) with model output (SHAP log-odds values). The color gradient in panel (**c**) encodes DWI radiomics model-predicted probability, whereas that in panel (**d**) represents VSR.

SHAP analysis characterized global and individual contributions of each variable to the combined model. In the global bar plot (Figure 5a), variables were ranked according to their mean absolute SHAP values on the log-odds scale. VSR had the largest mean contribution (1.51), followed by DWI radiomics model-predicted probability, NLR, IMATI, VATI, CEA, and HRT2WI radiomics model-predicted probability. The beeswarm plot (Figure 5b) shows the distribution of feature values across patients and the direction of their contributions to the model output. In the dependence plots (Figure 5c,d), higher VSR and higher DWI radiomics model-predicted probability were associated with higher SHAP values, indicating greater contributions to the model-predicted risk of malignant transformation. These visualizations describe associations learned by the model and should not be interpreted as evidence of causality.

**Figure 6:**
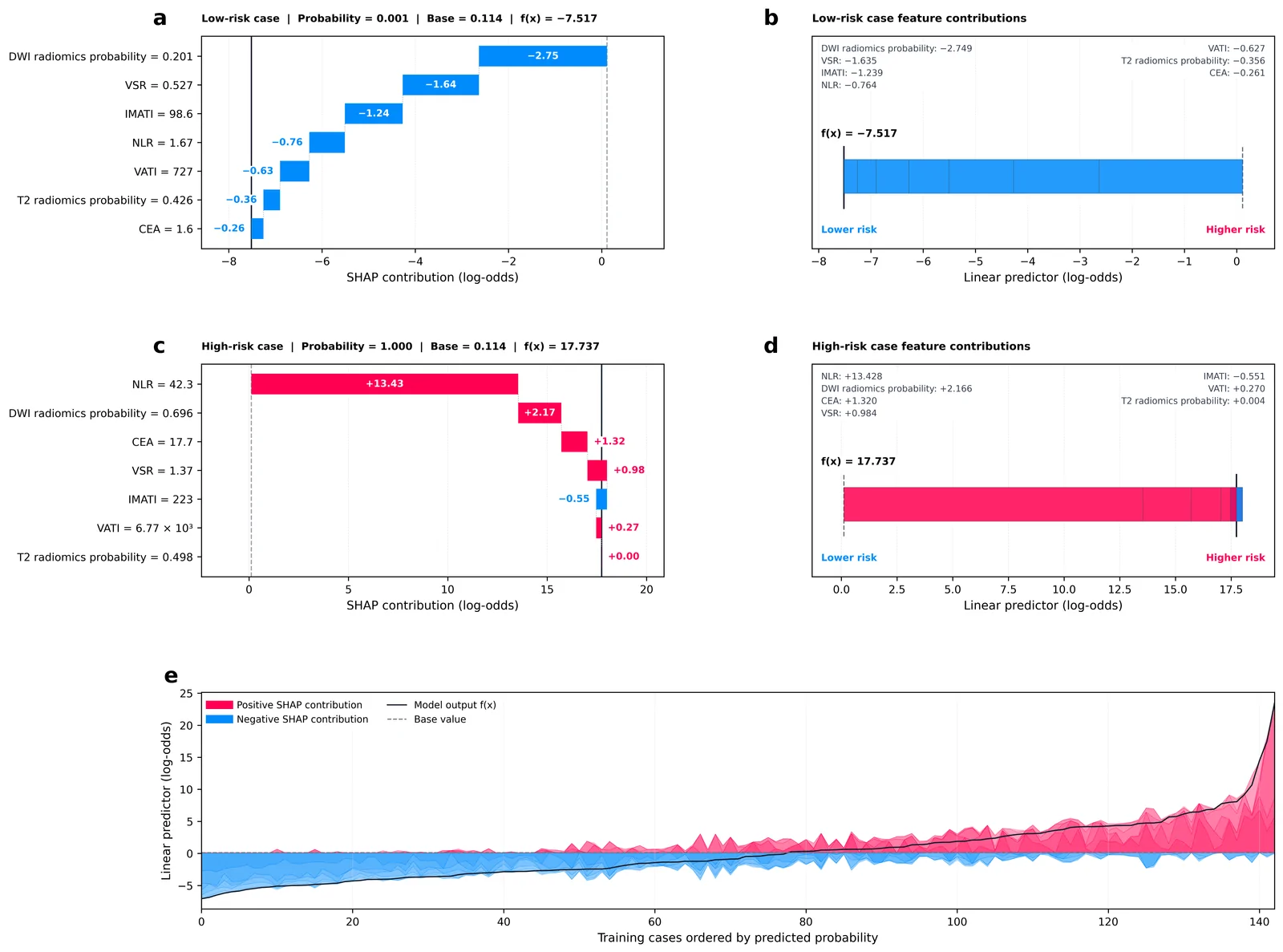
Local SHAP-derived interpretability of the multimodal prediction model. (**a**,**b**) Force plot for a representative low-risk patient. Blue bars indicate features with negative SHAP contributions that reduce the predicted log-odds of malignant transformation. (**c**,**d**) Force plot for a representative high-risk patient. Red bars indicate features with positive SHAP contributions that increase the predicted risk of malignant transformation. The base value denotes the mean model output across the cohort. (**e**) SHAP summary waterfall plot of cumulative feature contributions across training samples, ordered by model-predicted risk probability. Red and blue shaded regions correspond to positive and negative SHAP contributions to the linear predictor (log-odds), respectively.

For local interpretation, Figure 6a,b show a representative low-risk case. The final model-predicted probability was 0.001, compared with a base probability of 0.114. Negative SHAP contributions from DWI radiomics model-predicted probability, VSR, IMATI, NLR, and VATI lowered the model output. Figure 6c,d show a representative high-risk case with a model-predicted probability of 1.000. NLR had the largest positive SHAP contribution, followed by DWI radiomics model-predicted probability, CEA, VSR, IMATI, VATI, and HRT2WI radiomics model-predicted probability. Figure 6e visualizes cumulative feature contributions across the training samples. The final combined logistic regression score was also displayed as a nomogram incorporating CEA, NLR, IMATI, VATI, VSR, and the HRT2WI and DWI radiomics model-predicted probabilities. The nomogram provides a graphical representation of the model score to support preoperative risk stratification rather than a validated estimate of an individual’s absolute risk (Supplementary Figure S9).

## 4. Discussion

In this retrospective two-center study, we developed and externally validated a multimodal model that integrated HRT2WI and DWI radiomics model-predicted probabilities with CEA, NLR, and body composition predictors for preoperative risk stratification of malignant transformation in RAs. Logistic regression showed the most consistent performance among the five evaluated radiomics classifiers and was used for the combined model. In the external validation cohort, the combined model achieved an AUC of 0.867, higher than the observed AUCs of the component models. In an exploratory comparison involving one junior and one senior radiologist, the model had a higher AUC than the junior reader, whereas the difference from the senior reader was not statistically significant. These single-reader comparisons do not establish that the model outperforms radiologists in general. From a healthcare perspective, integrating imaging, laboratory, and body composition information may provide an additional signal for preoperative assessment. For patients with a higher model score, this signal could prompt closer review of endoscopic, biopsy, and imaging findings or multidisciplinary discussion when findings are discordant. Such patient benefit remains a possibility rather than a demonstrated effect.

We compared five ML algorithms—LR, SVM, RF, XGBoost, and LightGBM—to construct HRT2WI and DWI radiomics models. LR showed the most consistent performance across cohorts for both HRT2WI (AUCs, 0.716, 0.617, and 0.691) and DWI radiomics models (AUCs, 0.831, 0.750, and 0.628) in the training, internal validation, and external validation cohorts, respectively. Tree-based ensemble algorithms, including XGBoost and LightGBM, achieved higher AUCs in the training cohort but showed greater performance declines in external validation, whereas LR showed more consistent performance across cohorts. This observation is consistent with recent rectal MRI radiomics studies suggesting that complex ensemble models may be susceptible to overfitting in moderate sample sizes [27]. Other multi-algorithm comparative studies have also reported lower prediction variability and better resistance to overfitting with LR after feature reduction [28,29]. Unlike tree-based classifiers, LR is intrinsically interpretable and facilitates subsequent SHAP-based model interpretation [30]. Our findings add to the limited literature on rectal adenomas. Li et al. [8] analyzed 53 patients and reported test cohort AUCs of 0.867 for a combined LR model and 0.900 for a combined SVM model. Jia et al. [23] reported an AUC of 0.927 for a biexponential intravoxel incoherent motion model in 60 patients; neither study included an independent external cohort. The lower external AUCs of our individual HRT2WI and DWI signatures than those in earlier single-center studies may reflect the stricter two-center evaluation, differences in acquisition and preprocessing, and biological heterogeneity. The combined model’s AUC decreased from 0.956 in the training cohort to 0.867 in the external validation cohort, an absolute reduction of 0.089. The corresponding reduction for the body composition model was 0.120 (0.929 to 0.809). These declines indicate that training performance should be interpreted cautiously and are compatible with development-related optimism and reduced transportability. However, the differences cannot be attributed solely to overfitting, because the external cohort also differed in case mix, malignant transformation prevalence, and MRI acquisition conditions. The training-to-external AUC differences therefore describe performance attenuation rather than isolate the magnitude of overfitting. These findings underscore the importance of prioritizing external cohort generalizability rather than training set performance alone when selecting radiomics algorithms. Sensitivity analyses provided a more conservative assessment of model development. When feature selection, tuning, and classifier selection were repeated within nested cross-validation, HRT2WI discrimination was attenuated, whereas DWI retained moderate discrimination. Alternative Elastic Net clinical and body composition models and a simpler four-probability fusion model were also evaluated. These findings highlight the contribution of body composition information while cautioning against interpreting the original HRT2WI results or a particular fusion specification as unequivocally stable.

In addition to radiomic features, systemic inflammatory markers and body composition can reflect host nutritional and metabolic status and are regarded as key modulators of cancer-related prognostic outcomes [31,32]. The body composition findings show that host metabolic phenotype may carry more information about malignant transformation than BMI alone. In multivariable analysis, high IMATI, VATI, and VSR were independently associated with malignant transformation. Accordingly, the body composition model achieved an external AUC of 0.809, higher than the clinical model (0.668), HRT2WI radiomics model (0.691), and DWI radiomics model (0.628). These results are consistent with epidemiologic and quantitative CT studies linking visceral adiposity to colorectal adenoma and advanced adenoma [19,32]. VAT is metabolically active and can promote insulin resistance, chronic low-grade inflammation, and secretion of cytokines such as interleukin-6 and tumor necrosis factor alpha; these processes may support epithelial proliferation, immune dysregulation, and a microenvironment permissive for malignant progression [33,34]. These metabolic and inflammatory pathways may plausibly contribute to adenoma malignant transformation, although this observational study cannot establish biological mechanisms or causality. VSR may be informative because it captures the balance between pro-inflammatory visceral fat and the predominantly energy storage role of subcutaneous fat, while IMATI may reflect ectopic lipid deposition and impaired muscle metabolic function [35]. The absence of BMI as an independent predictor in our cohort reinforces the limitation of a single anthropometric index when tissue distribution differs between individuals. Previous studies have demonstrated that CEA and CA19-9 are independent prognostic risk factors in patients with colorectal cancer [36,37]. The elevated levels of CEA and CA19-9 are associated with poorer overall survival and recurrence-free survival. NLR reflects host tumor-promoting inflammatory status and has proven prognostic value in colorectal neoplasms [38]. Importantly, these variables were measured before surgery and are widely available, so their integration with MRI signatures can improve risk stratification without adding an invasive test. Integrating body composition and laboratory biomarkers captures both tumor intrinsic heterogeneity and host systemic risk factors, partially accounting for the improved predictive performance of our combined model.

Interpretability was assessed at global and individual levels. The logistic regression framework expresses each component score in relation to the model log-odds, and SHAP decomposes the model output into feature-level contributions. Globally, VSR and DWI radiomics model-predicted probability had the largest mean absolute SHAP contributions. The dependence plots described the associations between these predictors and the model output and should not be interpreted as evidence of causality. This case-level decomposition can be more actionable than a single AUC because it allows for a clinician to identify which modifiable or confirmable factors are driving an individual estimate and to discuss the result in a multidisciplinary setting [39]. They may improve transparency of model use and facilitate discussion of factors contributing to a model-derived risk stratum, but they do not establish treatment benefit or replace multidisciplinary clinical assessment. The nomogram provides a compact graphical representation of the final model score for preoperative risk stratification. As reported in prior interpretable multimodal work [40], SHAP visualizations make model outputs traceable rather than opaque numerical results, which helps build clinician confidence and promotes translational application.

This study has several limitations. First, its retrospective design may have introduced selection bias. The cohort comprised patients who underwent surgery and had postoperative pathologic confirmation, and lesions smaller than 10 mm were excluded. The observed prevalence and model performance may therefore not generalize to screening populations, patients managed nonoperatively, or small rectal lesions. Second, the training cohort included 143 patients with 65 malignant transformation events, and the external validation cohort included 155 patients with 91 malignant transformation events. The higher prevalence of malignant transformation observed in the external validation cohort suggests spectrum differences that may affect model transportability to other patient populations. With a moderate event count, sequential variable selection procedures carry risk of model instability and overfitting. The bootstrap resampling was used to evaluate feature selection robustness, but instability cannot be fully excluded. Calibration curve estimates in validation cohorts show visible fluctuation, which is expected given limited sample size; quantitative calibration metrics are provided to complement graphical inspection. Third, variation in MRI acquisition parameters across centers may have affected radiomic measurements and model transportability to other institutions. Fourth, the reader comparison included only one junior and one senior radiologist and was not a multi-reader multi-case or AI-assisted study; these exploratory results cannot be generalized to broader radiologist populations, and it should therefore be regarded as exploratory rather than as evidence of a generalizable reader-level benefit. Collectively, these constraints indicate that the model cannot be directly deployed into routine clinical practice at present. Prospective multicenter studies with multi-reader evaluation and assessment of clinical impact are needed. Finally, SHAP provides model-based explanations rather than direct biological validation of individual radiomic features. Taken together, these considerations reinforce that prospective external multicenter evaluation remains essential before any real world clinical application of this multimodal risk stratification model.

## 5. Conclusions

In conclusion, this interpretable multimodal model showed some discriminatory ability for preoperative risk stratification of malignant transformation in surgically treated RAs. The model may provide an additional risk signal to help identify patients who warrant further diagnostic assessment or multidisciplinary discussion within routine care. It is not a substitute for endoscopic assessment, pathologic diagnosis, or treatment decisions. Prospective multicenter validation, multi-reader assessment, and evaluation of clinical impact are required before the model can be considered for routine clinical use.

## Figures and Tables

**Table 1:** Comparison of the present study with representative previous studies.

Study	Study Population	Imaging and Modeling Approach	Key result and Validation	Innovation and Limitations
Li et al. [8]	53 patients; training cohort, *n* = 42; test cohort, *n* = 11	HRT2WI and DWI radiomics; logistic regression and support vector machine models	Combined-model test AUCs: 0.867 (logistic regression) and 0.900 (support vector machine).	Evaluated combined HRT2WI–DWI radiomics for this endpoint; limited by an 11-patient test set and no independent external validation.
Jia et al. [23]	60 patients with pathology-confirmed rectal adenoma, with or without canceration	IVIM-DWI parameters derived from mono-exponential, bi-exponential, and stretched exponential models	Bi-exponential model AUC: 0.927 (95% CI, 0.829–0.978).	Compared quantitative diffusion models for this endpoint; limited by the small cohort and no reported independent external validation.
Present study	359 surgically treated patients from two centers	HRT2WI and DWI radiomics model-predicted probabilities integrated with clinical and CT body composition predictors	Combined-model external validation AUC: 0.867 (95% CI, 0.803–0.919).	Integrated tumor imaging and host-related predictors with external validation; limited by retrospective surgical selection, suboptimal calibration, and untested clinical impact.

**Table 2:** Comparison of clinical characteristics in the training, internal and external validation cohorts.

Characteristic	Training Cohort (*n* = 143)				Internal Cohort (*n* = 61)				External Cohort (*n* = 155)				*p-Inter*	*q-Inter*
	RA (*n* = 78)	Malignant (*n* = 65)	*p*	*q*	RA (*n* = 33)	Malignant (*n* = 28)	*p*	*q*	RA (*n* = 64)	Malignant (*n* = 91)	*p*	*q*		
Age (years)	65.50 [57.25, 70.00]	67.00 [60.00, 74.00]	0.112	0.247	63.48 ± 11.83	67.82 ± 11.71	0.157	0.696	62.89 ± 10.28	64.49 ± 11.60	0.366	0.540	0.574	0.818
Sex			0.312	0.572			0.471	0.802			0.221	0.375	0.349	0.640
Male	39 (50.0)	38 (58.5)			17 (51.5)	17 (60.7)			36 (56.2)	60 (65.9)				
Female	39 (50.0)	27 (41.5)			16 (48.5)	11 (39.3)			28 (43.8)	31 (34.1)				
Hypertension			0.951	1.000			0.921	0.965			0.912	0.916	0.317	0.633
No	44 (56.4)	37 (56.9)			22 (66.7)	19 (67.9)			36 (56.2)	52 (57.1)				
Yes	34 (43.6)	28 (43.1)			11 (33.3)	9 (32.1)			28 (43.8)	39 (42.9)				
Diabetes			0.837	0.969			0.384	0.802			0.457	0.628	0.043 *	0.143
No	70 (89.7)	59 (90.8)			24 (72.7)	23 (82.1)			52 (81.2)	78 (85.7)				
Yes	8 (10.3)	6 (9.2)			9 (27.3)	5 (17.9)			12 (18.8)	13 (14.3)				
Smoking history			0.500	0.789			0.720	0.875			0.005 *	0.029 *	0.046 *	0.143
No	69 (88.5)	55 (84.6)			29 (87.9)	23 (82.1)			56 (87.5)	62 (68.1)				
Yes	9 (11.5)	10 (15.4)			4 (12.1)	5 (17.9)			8 (12.5)	29 (31.9)				
^a^ Alcohol use			0.753	0.920			0.653	0.875			<0.001 *	0.013 *	0.033 *	0.143
No	67 (85.9)	57 (87.7)			31 (93.9)	25 (89.3)			59 (92.2)	63 (69.2)				
Yes	11 (14.1)	8 (12.3)			2 (6.1)	3 (10.7)			5 (7.8)	28 (30.8)				
Family history			1.000	1.000			1.000	1.000			0.021 *	0.067	0.207	0.455
No	75 (96.2)	62 (95.4)			33 (100.0)	28 (100.0)			64 (100.0)	83 (91.2)				
Yes	3 (3.8)	3 (4.6)			0 (0.0)	0 (0.0)			0 (0.0)	8 (8.8)				
BMI (kg/m^2^)	23.58± 3.60	23.25± 2.98	0.550	0.789	23.60 ± 3.15	22.75 ± 3.00	0.286	0.802	23.62 ± 3.55	23.12 ± 3.17	0.368	0.540	0.894	0.945
CEA (ng/mL)	2.29 [1.62, 3.51]	3.06 [2.14, 4.87]	0.004 *	0.028 *	2.04 [1.62, 3.16]	3.06 [2.06, 5.07]	0.023 *	0.507	2.90 [2.10, 6.90]	5.70 [2.90, 10.95]	0.002 *	0.016 *	<0.001 *	<0.001 *
CA19-9 (IU/mL)	9.56 [6.06, 15.92]	14.57 [10.00, 21.75]	<0.001 *	0.006 *	8.68 [5.53, 13.83]	12.25 [6.35, 17.20]	0.130	0.696	6.90 [4.17, 11.88]	5.40 [2.65, 18.75]	0.554	0.687	<0.001 *	<0.001 *
CA125 (U/mL)	9.43 [7.59, 11.88]	9.82 [7.11, 11.93]	0.524	0.789	9.34 [6.97, 15.75]	10.91 [7.62, 13.13]	0.756	0.875	9.65 [7.12, 14.08]	9.80 [6.20, 14.55]	0.635	0.736	0.595	0.818
WBC (10^9^/L)	5.75 [5.03, 6.67]	5.90 [4.90, 7.30]	0.594	0.789	6.10 [5.00, 7.00]	6.35 [4.90, 8.60]	0.690	0.875	5.80 [4.80, 7.03]	6.10 [4.90, 7.45]	0.562	0.687	0.563	0.818
RBC (10^12^/L)	4.40 [4.07, 4.66]	4.38 [3.99, 4.63]	0.188	0.377	4.40± 0.52	4.30± 0.58	0.462	0.802	4.46 [4.16, 4.69]	4.36 [3.71, 4.73]	0.044 *	0.109	0.976	0.976
Hemoglobin (g/L)	136.50 [128.00, 146.75]	135.00 [119.00, 141.00]	0.048 *	0.150	133.00 [121.00, 146.00]	141.50 [122.50, 149.00]	0.902	0.965	137.50 [128.00, 148.25]	132.00 [121.00, 142.50]	0.033 *	0.092	0.883	0.945
Platelet count (10^9^/L)	192.49± 48.00	192.83± 64.89	0.972	1.000	194.00 [162.00, 225.00]	180.00 [152.50, 226.50]	0.669	0.875	184.00 [155.25, 215.00]	209.00 [160.00, 240.00]	0.076	0.151	0.796	0.945
Neutrophil count (10^9^/L)	3.75 [2.79, 4.30]	3.82 [3.20, 5.10]	0.103	0.247	3.87 [2.80, 4.82]	4.28 [3.25, 5.96]	0.434	0.802	3.67 [2.79, 4.30]	3.28 [1.85, 4.75]	0.158	0.290	0.009 *	0.065
Lymphocyte count (10^9^/L)	1.59 [1.20, 1.96]	1.26 [0.98, 1.65]	0.008 *	0.037 *	1.39 [1.24, 2.09]	1.44 [1.07, 1.71]	0.301	0.802	1.60 [1.27, 1.98]	1.37 [1.16, 1.62]	0.013 *	0.058	0.882	0.945
Monocyte count (10^9^/L)	0.35 [0.27, 0.42]	0.37 [0.30, 0.50]	0.089	0.244	0.34 [0.28, 0.39]	0.33 [0.29, 0.50]	0.643	0.875	0.36 [0.29, 0.44]	0.31 [0.21, 0.42]	0.049 *	0.109	0.132	0.362
NLR	2.29 [1.66, 3.12]	3.17 [2.03, 5.61]	0.005 *	0.028 *	2.33 [1.69, 3.68]	3.03 [2.12, 5.49]	0.158	0.696	2.08 [1.67, 2.97]	2.19 [1.33, 3.48]	0.916	0.916	0.021 *	0.113
PLR	121.96 [96.40, 162.90]	142.86 [115.71, 196.30]	0.025 *	0.091	134.72 [91.84, 162.50]	129.04 [109.03, 171.49]	0.474	0.802	114.83 [93.96, 158.48]	150.93 [114.43, 185.22]	0.002 *	0.016 *	0.902	0.945
LMR	4.36 [3.28, 6.32]	3.43 [2.25, 4.67]	<0.001 *	0.007 *	4.40 [3.32, 6.85]	3.15 [2.61, 5.83]	0.068	0.696	4.39 [3.43, 6.39]	4.68 [2.94, 6.61]	0.850	0.916	0.162	0.395
HPR	0.73 [0.65, 0.84]	0.70 [0.55, 0.91]	0.609	0.789	0.71 [0.58, 0.93]	0.75 [0.62, 0.82]	0.465	0.802	0.74 [0.61, 0.88]	0.65 [0.54, 0.81]	0.021 *	0.067	0.516	0.818
SMA (cm^2^)	108.30 [95.34, 135.40]	117.70 [93.08, 145.00]	0.374	0.374	109.70 [92.49, 134.80]	119.15 [93.67, 157.07]	0.343	0.490	113.95 [100.28, 137.65]	115.30 [97.89, 139.25]	0.796	0.796	0.705	0.959
SMD (HU)	33.99± 6.94	31.49± 7.58	0.043 *	0.063	35.89± 7.68	31.99± 4.75	0.019 *	0.031 *	35.90± 6.39	34.90± 7.77	0.382	0.477	0.016	0.158
IMAT (cm^2^)	5.82 [4.68, 7.83]	8.31 [6.31, 10.49]	<0.001 *	<0.001 *	5.48 [4.18, 7.11]	7.79 [5.52, 9.86]	0.004 *	0.009 *	5.72 [4.26, 7.77]	7.17 [5.49, 9.44]	<0.001 *	0.002 *	0.507	0.959
VAT (cm^2^)	71.07 [32.72, 108.28]	158.60 [114.60, 206.00]	<0.001 *	<0.001 *	75.65 [42.17, 125.40]	143.35 [90.55, 193.00]	<0.001 *	<0.001 *	72.00 [36.67, 101.60]	155.30 [122.55, 201.55]	<0.001 *	<0.001 *	0.716	0.959
SAT (cm^2^)	105.05 [65.24, 141.82]	116.50 [89.09, 151.00]	0.083	0.103	125.10 [72.30, 158.20]	113.25 [74.32, 141.57]	0.734	0.734	102.85 [74.09, 141.23]	119.60 [90.60, 146.45]	0.083	0.138	0.862	0.959
SMI (cm^2^/m^2^)	4560.38 [3882.19, 5365.46]	4933.50 [4189.08, 5792.25]	0.044 *	0.063	4760.60 [3890.55, 5443.04]	5143.34 [4125.38, 5888.48]	0.397	0.496	4530.70 [4144.53, 5219.65]	4658.41 [4110.45, 5499.12]	0.748	0.796	0.619	0.959
IMATI (cm^2^/m^2^)	249.01 [181.90, 341.37]	343.98 [253.81, 506.44]	<0.001 *	<0.001 *	250.14 [170.04, 286.99]	323.52 [227.20, 463.74]	0.007 *	0.013 *	223.78 [173.03, 314.18]	283.89 [211.10, 354.30]	0.005 *	0.009 *	0.161	0.804
VATI (cm^2^/m^2^)	2912.87 [1408.28, 4350.55]	6555.51 [4875.82, 8411.26]	<0.001 *	<0.001 *	2965.54 [1830.04, 5260.21]	6063.53 [4179.49, 8085.10]	<0.001 *	<0.001 *	2633.15 [1477.38, 4120.75]	6027.78 [4707.16, 7867.63]	<0.001 *	<0.001 *	0.959	0.959
SATI (cm^2^/m^2^)	4577.97 [2395.91, 6375.05]	4878.68 [3638.55, 6245.32]	0.098	0.109	4969.33 [2696.93, 6725.52]	4455.92 [2728.29, 6292.57]	0.691	0.734	4206.63 [2730.07, 6078.23]	4623.61 [3477.13, 6170.65]	0.198	0.282	0.928	0.959
VSR	0.65 [0.48, 0.91]	1.37 [1.05, 1.73]	<0.001 *	<0.001 *	0.62 [0.44, 0.92]	1.45 [1.04, 2.03]	<0.001 *	<0.001 *	0.62 [0.42, 0.83]	1.24 [0.98, 1.74]	<0.001 *	<0.001 *	0.727	0.959

Values are presented as mean ± SD, median [Q1, Q3], or *n* (%). *p* values compare RA and malignant transformation groups within each cohort using Welch’s t, Mann–Whitney U, Pearson χ2, or Fisher’s exact tests, as appropriate. *q* values are Benjamini–Hochberg FDR-adjusted *p* values. *p-inter* compares the three cohorts using Welch’s ANOVA, Kruskal–Wallis, or categorical tests, as appropriate. All tests were two-sided; * *p* < 0.05 indicates nominal significance, and * *q* < 0.05 indicates significance after multiplicity adjustment. ^a^ The non-drinking group was defined as individuals with complete abstinence from alcohol for at least the prior 1 year; drinkers included all participants with any alcohol consumption within this period drinking. RA = rectal adenoma; BMI = body mass index; CEA = carcinoembryonic antigen; CA19-9/CA125 = carbohydrate antigen 19-9/125; WBC/RBC = white/red blood cell count; NLR = neutrophil-to-lymphocyte ratio; PLR = platelet-to-lymphocyte ratio; LMR = lymphocyte-to-monocyte ratio; HPR = hemoglobin-to-platelet ratio. SMA = skeletal muscle area; SMD = skeletal muscle radiodensity; IMAT = intermuscular adipose tissue area; VAT = visceral adipose tissue area; SAT = subcutaneous adipose tissue area; SMI, IMATI, VATI, SATI = respective area indices normalized by squared lumbar height; VSR = visceral-to-subcutaneous fat ratio; HU = Hounsfield units.

**Table 3:** Results of univariate and multivariable logistic regression analyses of clinical factors in the training cohort.

Clinical Factors	Univariate Analysis		Multivariable Analysis	
	OR (95% CI)	*p* Value	OR (95% CI)	*p* Value
Sex (female vs. male)	0.711 (0.366–1.380)	0.313		
Age (years)	1.020 (0.990–1.051)	0.196		
Hypertension (yes vs. no)	0.979 (0.504–1.903)	0.951		
Diabetes (yes vs. no)	0.890 (0.292–2.710)	0.837		
Smoking (yes vs. no)	1.394 (0.530–3.669)	0.501		
Alcohol use (yes vs. no)	0.855 (0.322–2.270)	0.753		
Family history (yes vs. no)	1.210 (0.236–6.207)	0.820		
BMI (kg/m^2^)	0.970 (0.878–1.072)	0.554		
CEA (ng/mL)	1.222 (1.057–1.413)	0.007 *	1.228 (1.055–1.429)	0.008 *
CA19-9 (IU/mL)	1.040 (1.011–1.070)	0.007 *		
CA125 (U/mL)	1.009 (0.982–1.038)	0.511		
WBC (×10^9^/L)	1.150 (0.981–1.348)	0.084		
RBC (×10^12^/L)	0.516 (0.265–1.004)	0.051		
Hemoglobin (g/L)	0.976 (0.956–0.995)	0.016 *		
Platelet count (×10^9^/L)	1.000 (0.994–1.006)	0.971		
Neutrophil count (×10^9^/L)	1.263 (1.040–1.535)	0.019 *		
Lymphocyte count (×10^9^/L)	0.489 (0.270–0.886)	0.018 *		
Monocyte count (×10^9^/L)	11.246 (1.352–93.561)	0.025 *		
NLR	1.240 (1.062–1.447)	0.007 *	1.244 (1.059–1.463)	0.008 *
PLR	1.007 (1.001–1.013)	0.018 *		
LMR	0.795 (0.674–0.938)	0.007 *		
HPR	1.185 (0.368–3.817)	0.776		

The final locked model retained CEA and NLR after prespecified screening, collinearity assessment, and backward elimination. OR = odds ratio; CI = confidence interval (lower to upper limit); BMI = body mass index; CEA = carcinoembryonic antigen; CA19-9 = carbohydrate antigen 19-9; CA125 = carbohydrate antigen 125; WBC = white blood cell count; RBC = red blood cell count; NLR = neutrophil-to-lymphocyte ratio; PLR = platelet-to-lymphocyte ratio; LMR = lymphocyte-to-monocyte ratio; HPR = hemoglobin-to-platelet ratio; * *p* < 0.05.

**Table 4:** Univariable and multivariable logistic regression of body composition risk strata in the training cohort.

Body Composition	Univariate Analysis		Multivariable Analysis	
	OR (95% CI)	*p* Value	OR (95% CI)	*p* Value
SMA, high-risk group	2.246 (1.092–4.620)	0.028 *	2.068 (0.661–6.470)	0.212
SMD, high-risk group	2.246 (1.092–4.620)	0.028 *	0.804 (0.180–3.582)	0.775
IMAT, high-risk group	5.042 (2.451–10.370)	<0.001 *	-	
VAT, high-risk group	16.364 (7.097–37.729)	<0.001 *	1.196 (0.292–4.895)	0.803
SAT, high-risk group	5.911 (2.796–12.497)	<0.001 *	-	
SMI, high-risk group	2.614 (1.202–5.684)	0.015 *	3.566 (0.895–14.206)	0.071
IMATI, high-risk group	5.750 (2.697–12.257)	<0.001 *	6.785 (1.498–30.734)	0.013 *
VATI, high-risk group	53.224 (12.107–233.988)	<0.001 *	10.701 (1.274–89.909)	0.029 *
SATI, high-risk group	5.687 (2.764–11.701)	<0.001 *	3.471 (0.847–14.234)	0.084
VSR, high-risk group	19.735 (8.335–46.729)	<0.001 *	4.909 (1.043–23.092)	0.044 *

Dashes indicate variables removed before entry into the final multivariable model during collinearity reduction. OR = odds ratio; CI = confidence interval (lower to upper limit); SMA = skeletal muscle area; SMD = skeletal muscle density; IMAT = intermuscular adipose tissue area; VAT = visceral adipose tissue area; SAT = subcutaneous adipose tissue area; SMI = skeletal muscle index; IMATI = intermuscular adipose tissue index; SATI = subcutaneous adipose tissue index; VATI = visceral adipose tissue index; VSR = visceral-to-subcutaneous fat ratio; * *p* < 0.05.

**Table 5:** Comparison of the performance of the component and combined models in the three cohorts.

Models	AUC (95% CI)	Accuracy	Sensitivity	Specificity	PPV	NPV	DeLong *p* Value (vs Combined Model)
**Training cohort**							
Clinical	0.705 (0.623–0.788)	0.692	0.538	0.821	0.714	0.681	< 0.001 *
Body composition	0.929 (0.886–0.965)	0.860	0.923	0.808	0.800	0.926	0.172
HRT2WI radiomics	0.716 (0.625–0.797)	0.692	0.800	0.603	0.627	0.783	< 0.001 *
DWI radiomics	0.831 (0.759–0.897)	0.776	0.754	0.795	0.754	0.795	< 0.001 *
Combined	0.956 (0.922–0.981)	0.902	0.938	0.872	0.859	0.944	Ref
**Internal validation cohort**							
Clinical	0.669 (0.518–0.804)	0.672	0.500	0.818	0.700	0.659	< 0.001 *
Body composition	0.804 (0.674–0.910)	0.754	0.750	0.758	0.724	0.781	0.032 *
HRT2WI radiomics	0.617 (0.467–0.761)	0.557	0.750	0.394	0.512	0.650	< 0.001 *
DWI radiomics	0.750 (0.614–0.863)	0.721	0.607	0.818	0.739	0.711	0.024 *
Combined	0.908 (0.830–0.970)	0.836	0.821	0.848	0.821	0.848	ref
**External validation cohort**							
Clinical	0.668 (0.574–0.758)	0.632	0.648	0.609	0.702	0.549	< 0.001 *
Body composition	0.809 (0.740–0.875)	0.755	0.725	0.797	0.835	0.671	0.040 *
HRT2WI radiomics	0.691 (0.601–0.769)	0.677	0.681	0.672	0.747	0.597	< 0.001 *
DWI radiomics	0.628 (0.540–0.718)	0.548	0.396	0.766	0.706	0.471	< 0.001 *
Combined	0.867 (0.803–0.919)	0.729	0.626	0.875	0.877	0.622	ref

AUC = area under the curve; CI = confidence interval (lower to upper limit); NPV = negative predictive value; PPV = positive predictive value; HRT2WI = high-resolution T2-weighted imaging; DWI = diffusion-weighted imaging; Ref = reference. All listed *p* values are from paired DeLong test comparing each individual component model against the combined model within the same cohort; combined model serves as reference (ref). No pairwise comparisons among component models are shown in this table. * *p* < 0.05.

## Data Availability

The datasets generated or analyzed during the study are not publicly available because of Institutional Review Board restrictions related to participant confidentiality. They are available from the corresponding author on reasonable request. Code used for image preprocessing, feature extraction, model development, and statistical analysis is available from the corresponding author on reasonable request.

## Supplementary Materials

The following supporting information can be downloaded at: https://www.mdpi.com/article/10.3390/healthcare14213596/s1, Figure S1: Flowchart of patient enrollment. Figure S2: Measurement of body composition parameters. Figure S3: Receiver operating characteristic (ROC) curves and optimal cut off thresholds of body composition parameters in female. Figure S4: Receiver operating characteristic (ROC) curves and optimal cut off thresholds of body composition parameters in male. Figure S5: LASSO Regression Analysis and corresponding coefficients of retained feature of HRT2WI radiomics model. Figure S6: LASSO Regression Analysis and corresponding coefficients of retained feature of DWI radiomics model. Figure S7: Nomogram of combined model for preoperative prediction of malignant transformation in rectal adenoma. Figure S8: Feature selection frequencies across four model domains in 1000 bootstrap resamples. Figure S9: Discrimination, calibration, and decision-curve analyses of the clinical, body-composition, and four-probability fusion models in the sensitivity analysis. Table S1: MRI scanners information and scanning parameters of two hospitals. Table S2: Distribution of body composition risk strata across the three cohorts. Table S3: Comparison of the performance of five ML algorithms in HRT2WI radiomics model. Table S4: Comparison of the performance of five ML algorithms in DWI radiomics model. Table S5: AUCs of five radiomics algorithms from nested cross-validation and locked-model validation in HRT2WI and DWI. Table S6: Exploratory paired net benefit at illustrative risk thresholds.

## Abbreviations

The following abbreviations are used in this manuscript:

AUC	Area under the curve
BMI	Body mass index
CA19-9	Carbohydrate antigen 19-9
CA125	Carbohydrate antigen 125
CEA	Carcinoembryonic antigen
CI	Confidence interval
DCA	Decision curve analysis
DWI	Diffusion-weighted imaging
GLDM	Gray-level dependence matrix
GLCM	Gray-level co-occurrence matrix
GLRLM	Gray-level run-length matrix
GLSZM	Gray-level size-zone matrix
HPR	Hemoglobin-to-platelet ratio
HRT2WI	high-resolution T2-weighted imaging
ICC	Intraclass correlation coefficient
IMAT	Intermuscular adipose tissue
IMATI	Intermuscular adipose tissue index
LASSO	Least absolute shrinkage and selection operator
LightGBM	Light gradient boosting machine.
LMR	Lymphocyte-to-monocyte ratio
LR	Logistic regression
MRI	Magnetic resonance imaging
NGTDM	Neighboring gray-tone difference matrix
NLR	Neutrophil-to-lymphocyte ratio
NPV	Negative predictive value
OR	Odds ratio
PLR	Platelet-to-lymphocyte ratio
PPV	Positive predictive value
RBC	Red blood cell counts
ROI	Region of interest
RF	Random forest
SAT	Subcutaneous adipose tissue
SHAP	SHapley Additive exPlanations
SMD	Skeletal muscle density
SMI	Skeletal muscle index
SVM	Support vector machine
VAT	Visceral adipose tissue
VATI	Visceral adipose tissue index
VSR	Visceral-to-subcutaneous fat ratio
WBC	White blood cell counts
XGBoost	Extreme Gradient Boosting
