# Supplementary material for: An Interpretable Multimodal Model Integrating Multiparametric MRI Radiomics and Body Composition for Preoperative Risk Stratification of Malignant Transformation in Rectal Adenomas: A Two-Center Study
Source: Healthcare (Basel). 2026 Oct 23;14(21):3596. doi: 10.3390/healthcare14213596 (PMC13762873; doi:10.3390/healthcare14213596)
Supplement: Supplementary file 1 [file healthcare-14-03596-s001.zip › healthcare-4595752-supplementary/Supplementary Materials.docx]

**Supplementary materials**

1. Image preprocessing steps for radiomics feature extraction - *Pages 3*.

2. Supplementary Table S1. MRI scanners information and scanning parameters of two hospitals - *Pages 4*.

3. Supplementary Table S2. Distribution of body composition risk strata across the three cohorts - *Pages 5*.

4. Supplementary Table S3. Comparison of the performance of five ML algorithms in HRT2WI radiomics model - *Pages 6*.

5. Supplementary Table S4. Comparison of the performance of five ML algorithms in DWI radiomics model - *Pages 7*.

6. Supplementary Table S5. AUCs of five radiomics algorithms from nested cross-validation and locked-model validation in HRT2WI and DWI- *Pages 8*.

7.Supplementary Table S6. Exploratory paired net benefit at illustrative risk thresholds- *Pages 9*.

8. Supplementary Figure S1. Flowchart of patient enrollment. - *Pages 10*.

9. Supplementary Figure S2. Measurement of body composition parameters. - *Pages 11*.

10. Supplementary Figure S3. Receiver operating characteristic (ROC) curves and optimal cut off thresholds of body composition parameters in female. - *Pages 12*.

11. Supplementary Figure S4. Receiver operating characteristic (ROC) curves and optimal cut off thresholds of body composition parameters in male. - *Pages 13*.

12.Supplementary Figure S5. LASSO Regression Analysis and corresponding coefficients of retained feature of HRT2WI radiomics model. - *Pages 14*.

13.Supplementary Figure S6. LASSO Regression Analysis and corresponding coefficients of retained feature of DWI radiomics model - *Pages 15*.

14.Supplementary Figure S7. Nomogram of combined model for preoperative prediction of malignant transformation in rectal adenoma - *Pages 16*.

15.Supplementary Figure S8. Feature selection frequencies across four model domains in 1,000 bootstrap resamples. - *Pages 17*.

16.Supplementary Figure S9. Discrimination, calibration, and decision-curve analyses of the clinical, body-composition, and four-probability fusion models in the sensitivity analysis- *Pages 18*.

**Image preprocessing steps for radiomics feature extraction**

**1.****HRT2****WI Images preprocessing steps**

HRT2 images and masks were resampled to a voxel spacing of 1×1×3 mm³ using SimpleITK BSpline interpolation for images and nearest-neighbor interpolation for masks. Image intensities were clipped to the 1^st^-99^th^ percentiles, after which each clipped image underwent z-score normalization. Original-image radiomic features were extracted using PyRadiomics (version 3.0.1) with normalize = false, resampledPixelSpacing = null, and binWidth = 0.25; thus, normalization and resampling were not repeated during PyRadiomics extraction. Wavelet and Laplacian-of-Gaussian filtered image families were not included.

**2.** **DWI Images preprocessing steps**

DWI images were resampled in plane to 1.5×1.5 mm^2^ while preserving the original through-plane spacing; linear interpolation was used for images and nearest-neighbor interpolation for masks. Foreground intensities were clipped to the 1st-99th percentiles and standardized within each image by z transformation, with background voxels set to zero. Original-image radiomics features were extracted using PyRadiomics (version 3.0.1) with a fixed bin width of 0.25, force2D = True, and force2Ddimension = 0.

| **Table S1** **MRI scanners information and scanning parameters of two hospitals** | | | | | | | | | |
| --- | --- | --- | --- | --- | --- | --- | --- | --- | --- |
| **Institution** | **Scanner** | **Sequence** | **TR**  **(ms)** | **TE**  **(ms** | **FOV (mm)** | **Matrix** | **Slice Thickness (mm)** | **Gap (mm)** | **Flip angle** |
| **Center 1** | GE Discovery MR750w (3.0T) | HRT2WI | 3800 | 120 | 160×160 | 320×256 | 3 | 0.3 | 111 |
|  |  | DWI | 5800 | 70 | 380×380 | 128×128 | 5 | 1 | 90 |
|  | GE Signa HDxt (3.0T) | HRT2WI | 3300 | 136 | 160×160 | 320×224 | 3 | 0.3 | 90 |
|  |  | DWI | 5900 | 66 | 250×250 | 130×90 | 5 | 1 | 90 |
|  | GE Signa Excite (3.0T) | HRT2WI | 3600 | 103 | 160×160 | 256×192 | 3 | 0.3 | 90 |
|  |  | DWI | 4300 | 75 | 300×300 | 128×128 | 5 | 1 | 90 |
|  | Siemens-Skyra (3.0T) | HRT2WI | 5800 | 99 | 160×160 | 320×349 | 3 | 0.3 | 120 |
|  |  | DWI | 4400 | 61 | 380×380 | 160×95 | 5 | 1 | 90 |
| **Center 2** | GE Discovery MR750w (3.0T) | HRT2WI | 3800 | 120 | 160×160 | 320×256 | 3 | 0.3 | 111 |
|  |  | DWI | 5800 | 70 | 380×380 | 128×128 | 5 | 1 | 90 |
|  | GE-Signa HDxt (3.0T) | HRT2WI | 3300 | 136 | 160×160 | 320×224 | 3 | 0.3 | 90 |
|  |  | DWI | 5900 | 66 | 250×250 | 130×90 | 5 | 1 | 90 |
|  | GE Signa Excite (3.0T) | HRT2WI | 3600 | 103 | 160×160 | 256×192 | 3 | 0.3 | 90 |
|  |  | DWI | 4300 | 75 | 300×300 | 128×128 | 5 | 1 | 90 |
|  | United Imaging  uMR 790 (3.0T) | HRT2WI | 4980 | 130 | 219×240 | 320×234 | 3 | 0.3 | 120 |
|  |  | DWI | 5738 | 67 | 300×300 | 128×128 | 5 | 1 | 90 |
| MRI = magnetic resonance imaging; TR = repetition time; TE = echo time; FOV = field of view; HRT2WI = high-resolution T2-weighted imaging; DWI = diffusion‑weighted imaging. | | | | | | | | | |

**Table S2 Distribution of body composition risk strata across the three cohorts**

| **Characteristic** | **Stratum** | **Training cohort (n = 143)** | **Internal validation (n = 61)** | **External validation (n = 155)** | ***p* value** |
| --- | --- | --- | --- | --- | --- |
| Sex | Women | 66 (46.15) | 27 (44.26) | 59 (38.06) | 0.349 |
|  | Men | 77 (53.85) | 34 (55.74) | 96 (61.94) |  |
| Outcome | Adenoma | 78 (54.55) | 33 (54.10) | 64 (41.29) | 0.048* |
|  | Malignant transformation | 65 (45.45) | 28 (45.90) | 91 (58.71) |  |
| SMA | Low risk | 55 (38.46) | 24 (39.34) | 60 (38.71) | 0.993 |
|  | High risk | 88 (61.54) | 37 (60.66) | 95 (61.29) |  |
| SMD | Low risk | 94 (65.73) | 40 (65.57) | 79 (50.97) | 0.019* |
|  | High risk | 49 (34.27) | 21 (34.43) | 76 (49.03) |  |
| IMAT | Low risk | 67 (46.85) | 32 (52.46) | 74 (47.74) | 0.756 |
|  | High risk | 76 (53.15) | 29 (47.54) | 81 (52.26) |  |
| VAT | Low risk | 71 (49.65) | 30 (49.18) | 62 (40.00) | 0.200 |
|  | High risk | 72 (50.35) | 31 (50.82) | 93 (60.00) |  |
| SAT | Low risk | 54 (37.76) | 22 (36.07) | 60 (38.71) | 0.936 |
|  | High risk | 89 (62.24) | 39 (63.93) | 95 (61.29) |  |
| SMI | Low risk | 41 (28.67) | 19 (31.15) | 57 (36.77) | 0.318 |
|  | High risk | 102 (71.33) | 42 (68.85) | 98 (63.23) |  |
| IMATI | Low risk | 59 (41.26) | 30 (49.18) | 70 (45.16) | 0.557 |
|  | High risk | 84 (58.74) | 31 (50.82) | 85 (54.84) |  |
| VATI | Low risk | 51 (35.66) | 22 (36.07) | 56 (36.13) | 0.996 |
|  | High risk | 92 (64.34) | 39 (63.93) | 99 (63.87) |  |
| SATI | Low risk | 50 (34.97) | 22 (36.07) | 58 (37.42) | 0.907 |
|  | High risk | 93 (65.03) | 39 (63.93) | 97 (62.58) |  |
| VSR | Low risk | 71 (49.65) | 29 (47.54) | 69 (44.52) | 0.673 |
|  | High risk | 72 (50.35) | 32 (52.46) | 86 (55.48) |  |

Variables are presented as frequency with percentages in parentheses. Sex-specific cutoffs were derived in the training cohort. High-risk and low-risk labels reflect the direction used in body composition modeling. *p* values were calculated using chi-square tests across the three cohorts. SMA = skeletal muscle area; SMD = skeletal muscle density; IMAT = intermuscular adipose tissue area; VAT = visceral adipose tissue area; SAT= subcutaneous adipose tissue area; SMI = skeletal muscle index; IMATI, intermuscular adipose tissue index; SATI, subcutaneous adipose tissue index; VATI, visceral adipose tissue index; VSR, visceral-to-subcutaneous fat ratio; **p* < 0.05.

**Table S3** **Comparison of the performance of five** **ML** **algorithms in HRT2WI radiomics model**

| **Cohort** | **ML algorithms** | **AUC (95% CI)** | **Accuracy** | **Sensitivity** | **Specificity** | **PPV** | **NPV** |
| --- | --- | --- | --- | --- | --- | --- | --- |
| Training | LR | 0.716 (0.629‑0.800) | 0.692 | 0.800 | 0.603 | 0.627 | 0.783 |
|  | SVM | 0.722 (0.634‑0.802) | 0.671 | 0.846 | 0.526 | 0.598 | 0.804 |
|  | RF | 0.835 (0.767‑0.895) | 0.748 | 0.862 | 0.654 | 0.675 | 0.850 |
|  | XGBoost | 0.784 (0.709‑0.851) | 0.706 | 0.846 | 0.590 | 0.632 | 0.821 |
|  | LightGBM | 0.786 (0.711‑0.859) | 0.706 | 0.831 | 0.603 | 0.635 | 0.810 |
| Internal validation | LR | 0.617 (0.477‑0.763) | 0.557 | 0.750 | 0.394 | 0.512 | 0.650 |
|  | SVM | 0.634 (0.493‑0.773) | 0.574 | 0.821 | 0.364 | 0.523 | 0.706 |
|  | RF | 0.606 (0.461‑0.752) | 0.574 | 0.679 | 0.485 | 0.528 | 0.640 |
|  | XGBoost | 0.601 (0.451‑0.742) | 0.525 | 0.679 | 0.394 | 0.487 | 0.591 |
|  | LightGBM | 0.610 (0.460‑0.748) | 0.541 | 0.750 | 0.364 | 0.500 | 0.632 |
| External validation | LR | 0.691 (0.606‑0.776) | 0.677 | 0.681 | 0.672 | 0.747 | 0.597 |
|  | SVM | 0.682 (0.587‑0.763) | 0.677 | 0.736 | 0.594 | 0.720 | 0.613 |
|  | RF | 0.651 (0.564‑0.734) | 0.652 | 0.681 | 0.609 | 0.713 | 0.574 |
|  | XGBoost | 0.689 (0.601‑0.775) | 0.658 | 0.780 | 0.484 | 0.683 | 0.608 |
|  | LightGBM | 0.675 (0.593‑0.760) | 0.632 | 0.747 | 0.469 | 0.667 | 0.566 |

ML= machine learning; HRT2WI = high-resolution T2-weighted imaging; LR = logistic regression; SVM = support vector machine; RF = random forest; XGBoost = Extreme Gradient Boosting; AUC = area under the curve; CI = confidence interval; PPV= positive predictive value; NPV= negative predictive value.

**Table S4 Comparison of the performance of five ML algorithms in DWI radiomics model**

| **Cohort** | **ML algorithms** | **AUC (95% CI)** | **Accuracy** | **Sensitivity** | **Specificity** | **PPV** | **NPV** |
| --- | --- | --- | --- | --- | --- | --- | --- |
| Training | LR | 0.831(0.760‑0.895) | 0.776 | 0.754 | 0.795 | 0.754 | 0.795 |
|  | SVM | 0.828(0.757‑0.892) | 0.783 | 0.754 | 0.808 | 0.766 | 0.797 |
|  | RF | 0.882(0.822‑0.933) | 0.818 | 0.708 | 0.910 | 0.868 | 0.789 |
|  | XGBoost | 0.923(0.878‑0.959) | 0.825 | 0.846 | 0.808 | 0.786 | 0.863 |
|  | LightGBM | 0.860(0.797‑0.915) | 0.769 | 0.785 | 0.756 | 0.729 | 0.808 |
| Internal validation | LR | 0.750(0.617‑0.869) | 0.721 | 0.607 | 0.818 | 0.739 | 0.711 |
|  | SVM | 0.759(0.624‑0.875) | 0.705 | 0.571 | 0.818 | 0.727 | 0.692 |
|  | RF | 0.711(0.571‑0.836) | 0.689 | 0.429 | 0.909 | 0.800 | 0.652 |
|  | XGBoost | 0.652(0.503‑0.786) | 0.557 | 0.500 | 0.606 | 0.519 | 0.588 |
|  | LightGBM | 0.671(0.526‑0.802) | 0.623 | 0.500 | 0.727 | 0.609 | 0.632 |
| External validation | LR | 0.628(0.534‑0.717) | 0.548 | 0.396 | 0.766 | 0.706 | 0.471 |
|  | SVM | 0.624(0.528‑0.713) | 0.535 | 0.363 | 0.781 | 0.702 | 0.463 |
|  | RF | 0.590(0.491‑0.683) | 0.413 | 0.077 | 0.891 | 0.500 | 0.404 |
|  | XGBoost | 0.605(0.507‑0.698) | 0.510 | 0.352 | 0.734 | 0.653 | 0.443 |
|  | LightGBM | 0.605(0.506‑0.698) | 0.516 | 0.385 | 0.703 | 0.648 | 0.446 |

ML= machine learning; HRT2WI = high-resolution T2-weighted imaging; LR = logistic regression; SVM = support vector machine; RF = random forest; XGBoost = Extreme Gradient Boosting; AUC = area under the curve; CI = confidence interval (lower to upper limit); PPV= positive predictive value; NPV= negative predictive value.

**Table S5 AUCs of five radiomics algorithms from nested cross-validation and locked-model validation in HRT2WI and DWI**

| **Cohort** | **ML algorithms** | **T2WI AUC (95% CI)** | **DWI AUC (95% CI)** |
| --- | --- | --- | --- |
| Training | LR | 0.624 (0.533–0.712) | 0.763 (0.684–0.836) |
|  | SVM | 0.535 (0.440–0.624) | 0.716 (0.634–0.796) |
|  | RF | 0.601 (0.500–0.691) | 0.792 (0.717–0.861) |
|  | XGBoost | 0.574 (0.482–0.664) | 0.792 (0.715–0.857) |
|  | LightGBM | 0.543 (0.449–0.647) | 0.799 (0.726–0.865) |
| Internal validation | LR | 0.510 (0.372–0.653) | 0.760 (0.629–0.874) |
|  | SVM | 0.535 (0.390–0.672) | 0.765 (0.633–0.877) |
|  | RF | 0.490 (0.350–0.641) | 0.696 (0.561–0.827) |
|  | XGBoost | 0.535 (0.386–0.676) | 0.662 (0.525–0.802) |
|  | LightGBM | 0.526 (0.371–0.687) | 0.703 (0.567–0.835) |
| External validation | LR | 0.692 (0.608–0.770) | 0.630 (0.539–0.714) |
|  | SVM | 0.691 (0.608–0.773) | 0.621 (0.531–0.707) |
|  | RF | 0.720 (0.632–0.804) | 0.613 (0.525–0.698) |
|  | XGBoost | 0.723 (0.642–0.796) | 0.572 (0.474–0.662) |
|  | LightGBM | 0.718 (0.637–0.796) | 0.612 (0.523–0.699) |

Training AUCs were calculated from pooled outer-fold predictions using five-fold outer and five-fold inner cross-validation. Within each outer training fold, feature selection and hyperparameter tuning were repeated within the corresponding training subsets; the outer held-out fold was used only for evaluation. Results are reported separately for each algorithm. Final models were fitted on the full training cohort and evaluated in the internal and external validation cohorts without further fitting or tuning.

ML= machine learning; HRT2WI = high-resolution T2-weighted imaging; LR = logistic regression; SVM = support vector machine; RF = random forest; XGBoost = Extreme Gradient Boosting; AUC = area under the curve; CI = confidence interval (lower to upper limit).

**Table S6 Exploratory paired net benefit at illustrative risk thresholds**

| **Cohort** | **Threshold** | **Combined NB** | **Difference vs all** | **Difference vs none** | **Difference vs clinical** | **Difference vs body** | **Difference vs T2** | **Difference vs DWI** |
| --- | --- | --- | --- | --- | --- | --- | --- | --- |
| Training | 0.10 | 0.421 (0.336, 0.505) | 0.027 (0.003, 0.046) | 0.421 (0.336, 0.505) | 0.027 (0.003, 0.046) | -0.003 (-0.030, 0.021) | 0.027 (0.003, 0.046) | 0.027 (0.003, 0.046) |
| Training | 0.20 | 0.407 (0.320, 0.493) | 0.089 (0.059, 0.115) | 0.407 (0.320, 0.493) | 0.089 (0.059, 0.115) | 0.007 (-0.024, 0.038) | 0.089 (0.059, 0.115) | 0.089 (0.059, 0.115) |
| Training | 0.30 | 0.397 (0.308, 0.483) | 0.176 (0.128, 0.221) | 0.397 (0.308, 0.483) | 0.159 (0.110, 0.209) | 0.023 (-0.019, 0.063) | 0.176 (0.128, 0.221) | 0.139 (0.091, 0.185) |
| Training | 0.40 | 0.371 (0.280, 0.459) | 0.280 (0.212, 0.345) | 0.371 (0.280, 0.459) | 0.217 (0.147, 0.287) | 0.023 (-0.026, 0.070) | 0.280 (0.212, 0.345) | 0.128 (0.070, 0.186) |
| Training | 0.50 | 0.343 (0.245, 0.441) | 0.434 (0.336, 0.531) | 0.343 (0.245, 0.441) | 0.224 (0.140, 0.308) | 0.035 (-0.028, 0.098) | 0.217 (0.133, 0.301) | 0.126 (0.056, 0.196) |
| Internal | 0.10 | 0.392 (0.266, 0.523) | -0.007 (-0.071, 0.044) | 0.392 (0.266, 0.523) | -0.007 (-0.071, 0.044) | -0.007 (-0.067, 0.049) | -0.007 (-0.071, 0.044) | -0.007 (-0.071, 0.044) |
| Internal | 0.20 | 0.377 (0.246, 0.512) | 0.053 (-0.020, 0.111) | 0.377 (0.246, 0.512) | 0.053 (-0.020, 0.111) | 0.045 (-0.016, 0.115) | 0.053 (-0.020, 0.111) | 0.053 (-0.020, 0.111) |
| Internal | 0.30 | 0.335 (0.204, 0.471) | 0.108 (0.005, 0.199) | 0.335 (0.204, 0.471) | 0.124 (0.030, 0.213) | 0.052 (-0.037, 0.141) | 0.108 (0.005, 0.199) | 0.066 (-0.037, 0.166) |
| Internal | 0.40 | 0.322 (0.186, 0.464) | 0.224 (0.098, 0.344) | 0.322 (0.186, 0.464) | 0.175 (0.071, 0.290) | 0.066 (-0.038, 0.169) | 0.240 (0.115, 0.361) | 0.169 (0.060, 0.279) |
| Internal | 0.50 | 0.279 (0.131, 0.426) | 0.361 (0.197, 0.525) | 0.279 (0.131, 0.426) | 0.164 (0.049, 0.295) | 0.049 (-0.066, 0.164) | 0.213 (0.066, 0.361) | 0.115 (0.000, 0.230) |
| External | 0.10 | 0.499 (0.416, 0.578) | -0.042 (-0.089, -0.001) | 0.499 (0.416, 0.578) | -0.042 (-0.089, -0.001) | 0.030 (-0.007, 0.069) | -0.042 (-0.089, -0.001) | -0.042 (-0.089, -0.001) |
| External | 0.20 | 0.452 (0.368, 0.531) | -0.032 (-0.092, 0.023) | 0.452 (0.368, 0.531) | -0.032 (-0.092, 0.023) | 0.018 (-0.029, 0.061) | -0.032 (-0.092, 0.023) | -0.034 (-0.094, 0.021) |
| External | 0.30 | 0.389 (0.306, 0.472) | -0.021 (-0.097, 0.051) | 0.389 (0.306, 0.472) | -0.021 (-0.098, 0.052) | -0.017 (-0.076, 0.040) | -0.021 (-0.097, 0.051) | 0.013 (-0.060, 0.084) |
| External | 0.40 | 0.333 (0.249, 0.417) | 0.022 (-0.075, 0.116) | 0.333 (0.249, 0.417) | 0.019 (-0.062, 0.099) | -0.037 (-0.105, 0.026) | 0.022 (-0.075, 0.116) | 0.108 (0.024, 0.189) |
| External | 0.50 | 0.277 (0.194, 0.361) | 0.103 (-0.019, 0.226) | 0.277 (0.194, 0.361) | 0.071 (-0.013, 0.155) | -0.058 (-0.135, 0.019) | 0.090 (-0.006, 0.181) | 0.187 (0.110, 0.265) |

NB = net benefit; CI = confidence interval(lower to upper limit); T2 = T2-weighted imaging; DWI = diffusion-weighted imaging. Differences are the combined model’s NB minus the comparator’s NB in the same patients; positive values favor the combined model. Parentheses show pointwise 95% CIs from 5,000 paired patient-level bootstrap resamples with fixed predictions. The thresholds of 0.10–0.50 were illustrative and were not clinically prespecified.

**Figure S1. Flowchart of patient enrollment.**

**Figure S2. Measurement of body composition parameters.** **(a-d)** Transverse non-contrast CT at the third lumbar vertebra (L3) level. **(a)** The areas of skeletal muscle were measured with tissue Hounsfield unit (HU) thresholds from −29 to +150 HU. **(b)** The areas of intermuscular adipose tissue were measured with tissue HU thresholds from −190 to −30 HU. **(c)** The areas of subcutaneous adipose tissue were measured with tissue HU thresholds from −190 to −30 HU. **(d)** The areas of visceral adipose tissue were measured with tissue HU thresholds from −150 to −50 HU.

**Figure S3. Receiver operating characteristic (ROC) curves and optimal cut‑off thresholds of body composition parameters in female.** **(a-j)** ROC curves of skeletal muscle area **(a)**, skeletal muscle density **(b)**, intermuscular adipose tissue area **(c)**, visceral adipose tissue area **(d)**, subcutaneous adipose tissue area **(e)**, skeletal muscle index **(f)**, intermuscular adipose tissue index **(g)**, visceral adipose tissue index **(h)**, subcutaneous adipose tissue index **(i)**, visceral-to-subcutaneous fat ratio **(j)**.

**Figure S4. Receiver operating characteristic (ROC) curves and optimal cut‑off thresholds of body composition parameters in male.** **(a-j)** ROC curves of skeletal muscle area **(a)**, skeletal muscle density **(b)**, intermuscular adipose tissue area **(c)**, visceral adipose tissue area **(d)**, subcutaneous adipose tissue area **(e)**, skeletal muscle index **(f)**, intermuscular adipose tissue index **(g)**, visceral adipose tissue index **(h)**, subcutaneous adipose tissue index **(i)**, visceral-to-subcutaneous fat ratio **(j)**.

**Figure S5.** **LASSO Regression Analysis and corresponding coefficients of retained feature of HRT2WI radiomics model.** **(a)** Coefficient‑profile plot showing the changing trajectories of feature coefficients against varying lambda (λ) values. Each coloured curve represents one radiomic feature. The optimal lambda (λ = 0.05116) is marked by the vertical dashed line. **(b)** Binomial deviance curve for cross‑validation. The red solid curve illustrates cross‑validation deviance, and the vertical dashed line denotes the optimal lambda value identified via 10‑fold cross‑validation. **(c)** Bar chart presenting the standardized coefficients of the seven final selected radiomic features included in the HRT2WI radiomic signature.

**Figure S6. LASSO Regression Analysis and corresponding coefficients of retained feature of DWI radiomics model**. **(a)** Coefficient‑profile plot showing the changing trajectories of feature coefficients against varying lambda (λ) values. Each coloured curve represents one radiomic feature. The optimal lambda (λ = 0.0438) is marked by the vertical dashed line. **(b)** Binomial deviance curve for cross‑validation. The red solid curve illustrates cross‑validation deviance, and the vertical dashed line denotes the optimal lambda value identified via 10‑fold cross‑validation. **(c)** Bar chart presenting the standardized coefficients of the seven final selected radiomic features included in the DWI radiomic signature.

**Figure S7. Nomogram of combined model for preoperative prediction of malignant transformation in rectal adenoma.** Feature values are converted to point scores; the sum of total points corresponds to the final predicted risk probability.

**Figure S8. Feature selection frequencies across four model domains in 1,000 bootstrap resamples.** (a) - (b) show the selection frequencies of the seven radiomic features retained in the original T2 and DWI models, respectively, under their corresponding feature-selection procedures. (c) - (d) show the five candidate variables for each of the clinical and body-composition Elastic Net models developed in the supplementary sensitivity analysis. These candidates were specified before the sensitivity analysis. Predictive performance of the two alternative models was evaluated using 5 × 5 nested cross-validation; the selection frequencies shown here were obtained from a separate analysis of 1,000 patient-level bootstrap resamples, with hyperparameter tuning and model fitting repeated in each resample. Selection frequency was calculated as the number of resamples in which a variable was selected divided by the number of successful resamples. Solid bars indicate variables retained in the corresponding model, whereas the hatched bar indicates a candidate variable not retained.

**Figure S9. Discrimination, calibration, and decision-curve analyses of the clinical, body-composition, and four-probability fusion models in the sensitivity analysis.** (a)-(c) show receiver operating characteristic curves in the training (n = 143; 65 events), internal validation (n = 61; 28 events), and external validation (n = 155; 91 events) cohorts, respectively. The legends report the area under the curve (AUC) and its 95% bootstrap confidence interval. (d)-(f) show calibration: points represent observed event rates in five groups defined by predicted probability, and error bars represent 95% Wilson confidence intervals. (g)-(i) show exploratory decision-curve analyses over threshold probabilities of 0.05-0.60. Shading around the fusion-model curve represents pointwise 95% confidence intervals from patient-level bootstrap resampling of fixed predictions.
